# LC-MS based metabolomic profiling for renal cell carcinoma histologic subtypes

**DOI:** 10.1038/s41598-019-52059-y

**Published:** 2019-10-30

**Authors:** Lun Jing, Jean-Marie Guigonis, Delphine Borchiellini, Matthieu Durand, Thierry Pourcher, Damien Ambrosetti

**Affiliations:** 1 Laboratory Transporter in Imaging and Radiotherapy in Oncology (TIRO), Institut de biosciences et biotechnologies d’Aix-Marseille (BIAM), Commissariat à lEnergie Atomique, Nice, France; 2 Laboratory Transporter in Imaging and Radiotherapy in Oncology (TIRO), school of medicine, Université Nice Sophia Antipolis, Université Côte d’Azur, Nice, France; 3 Medical Oncology Department, Centre Antoine Lacassagne, Nice, France; 4 Urology Department, Centre Hospitalier Universitaire, Nice, France; 5 Central Laboratory of Anatomopathology, Centre Hospitalier Universitaire, Nice, France

**Keywords:** Diagnostic markers, Renal cell carcinoma

## Abstract

Renal cell carcinomas (RCC) are classified according to their histological features. Accurate classification of RCC and comprehensive understanding of their metabolic dysregulation are of critical importance. Here we investigate the use of metabolomic analyses to classify the main RCC subtypes and to describe the metabolic variation for each subtype. To this end, we performed metabolomic profiling of 65 RCC frozen samples (40 clear cell, 14 papillary and 11 chromophobe) using liquid chromatography-mass spectrometry. OPLS-DA multivariate analysis based on metabolomic data showed clear discrimination of all three main subtypes of RCC (R^2^ = 75.0%, Q^2^ = 59.7%). The prognostic performance was evaluated using an independent cohort and showed an AUROC of 0.924, 0.991 and 1 for clear cell, papillary and chromophobe RCC, respectively. Further pathway analysis using the 21 top metabolites showed significant differences in amino acid and fatty acid metabolism between three RCC subtypes. In conclusion, this study shows that metabolomic profiling could serve as a tool that is complementary to histology for RCC subtype classification. An overview of metabolic dysregulation in RCC subtypes was established giving new insights into the understanding of their clinical behaviour and for the development of targeted therapeutic strategies.

## Introduction

Renal cell carcinoma (RCC) accounts for about 3% of malignancies in adults and 90–95% of all kidney cancers^1,2^. In its disseminated form, kidney cancer is an aggressive tumour and is one of the ten most frequent causes of cancer mortality^3,4^. RCCs are commonly classified according to the histologic features by which we distinguish three main subtypes: clear cell renal cell carcinoma (ccRCC) representing 70–75%, papillary renal cell carcinoma (papRCC) representing 10–15%, and chromophobe renal cell carcinoma (chroRCC) representing 5%^5^. This histological classification provides a primary level of information on the evolutionary risk of these tumours, with ccRCC being the most aggressive and metastatic subtype, and chroRCC being the most indolent^6,7^. Thus, the accurate diagnosis of the histologic subtype is important for prognosis and theranostic orientation^8^. Moreover, understanding of the biological origin responsible for the differences in clinical behaviour between RCC subtypes is crucial for the identification of appropriate targeted therapeutic strategies^9–11^. Thus, all analyses complementary to histology, including cytogenetics, immunohistochemistry, or metabolomics – as we will present here – are invaluable in RCC classification.

Metabolomics is the large-scale study of virtually all small molecules present within a cell, a tissue or a whole organism, and which provides a snapshot of all biochemical events occurring at the moment of the sample collection^12^. One of the hallmarks of cancer cells is the metabolic reprogramming that fuels their high energy needs^13^. In the last decade, untargeted metabolomic profiling, using liquid chromatography combined with mass spectrometry (LC-MS), has proven to be a promising tool in kidney cancer diagnostics and research. Previous MS-based metabolomic studies have already shown the possibility of discriminate RCC patients from healthy controls using tissue^14–19^, urine^17,20–22^ or serum^23^. Moreover, a recent study has also demonstrated the possibility of discrimination of different RCC stages, from 1 to 4, using combined metabolomic studies^24^. A RCC metabolic signature has been proposed in some of these studies involving essential pathways of energy metabolism: glycolysis, amino acid metabolism and fatty acid metabolism^25^. However, the vast majority of these studies were focused solely on clear cell RCCs and only a few studies^26,27^ investigated other RCC subtypes such as chroRCC or papRCC.

In this study, we performed the first untargeted metabolomic profiling analysis using LC-MS on all three main RCC histologic subtypes. We demonstrated that RCC subtypes could be accurately classified by multivariate analysis based on metabolomic data and we further identified the metabolic dysregulation in each subtype, with a focus on amino acids and acyl carnitines. These results provide new insights into the differences between oncogenesis mechanisms for different RCC subtypes and shows promise for the selection of efficient and adequate treatments and the discovery of new subtype-selective therapeutic targets.

## Results

### Metabolomic data analysis and metabolite identification

Thirty-seven randomly chosen frozen tissue samples (21 clear cell, 9 papillary and 7 chromophobe) were used as the training set for initial RCC subtype classification model development (Tables 1 and 2). The metabolites from each sample were extracted in parallel and analysed by LC-MS/MS. Raw spectrum data were then integrated in MZmine (Version 2.29) for chromatographic alignment and peak detection. 1591 and 898 peaks were isolated in positive and negative mode, respectively, and 852 (pos) and 469 (neg) could be identified in the human metabolome database. After elimination of the duplicates that were identified in both polarities, 1042 metabolites were selected for further analysis (Supplementary Dataset).

**Table 1:** Characteristics of the patients included in this study.

Cohort	Training set		Validation set	
	N	%	N	%
Number of Patients	37	—	28	—
Age at Surgery (YR)	64.3	—	65	—
**Gender**				
Male	27	73	19	68
Female	10	27	9	32
**Surgical Procedure**				
Radical nephrectomy	27	73	16	57
Partial nephrectomy	10	27	12	43
**Histology Subtype**				
Clear cell	21	57	19	68
Papillary	9	24	5	18
Chromophobe	7	19	4	14
**pT**				
1	15	41	16	57
2	4	11	2	7
3	18	48	10	36

**Table 2:** Grade distribution for ccRCC and papRCC.

Isup Grade	Training set		Validation set	
	N	%	N	%
2	8	27	10	42
3	13	43	11	46
4	9	30	3	13

For 8 of these RCC tumours (5 ccRCC and 3 chroRCC), we also collected adjacent normal tissue from the same nephrectomy surgical specimen and performed the same metabolomic profiling in order to determine the baseline of metabolic levels in healthy controls.

### Multivariate analysis for RCC subtype classification

**Figure 1:**
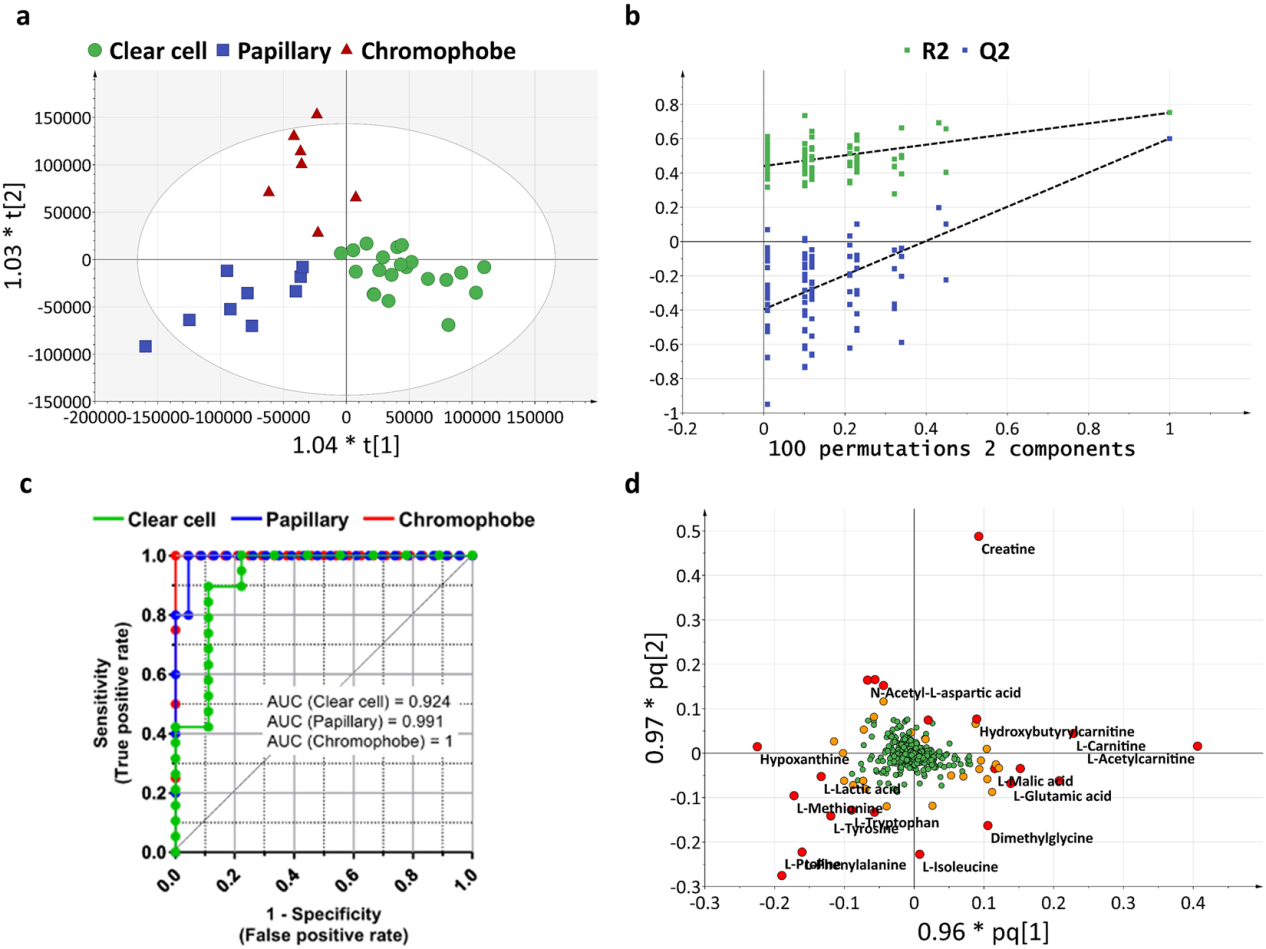
Renal cell carcinoma subtype classification based on untargeted metabolomics data. (**a**) OPLS-DA model of RCC subtype classification (N = 37). The model is composed of 2 predictive components and 1 orthogonal component and presents an R^2^X_(cum)_ of 44.2%, a goodness-of-fit R^2^ of 75.0%, a goodness-of-prediction Q^2^ of 59.7% and a CV-ANOVA p-value of 7.516 × 10^−8^. (**b**) Validation plot obtained from 100 permutation tests. (**c**) ROC (Receiver Operating Characteristic) curves obtained from an independent cohort (N = 28) showing the ability of OPLS-DA model to predict RCC subtypes. (**d**) Loading plot showing the most discriminative metabolites. The metabolites with VIP (Variable Importance for the Projection) >3 are highlighted with red circles; with VIP >2 are highlighted with orange circles. Variables with VIP >3 are used for further pathway analysis.

Raw metabolic data were mean-centred, and Pareto scaled before multivariate analysis. The Pareto scaling reduces the relative importance of high-intensity metabolites but preserves the integrity of the data structure compared to the classic unit-variant scaling^28^. Principal component analysis (PCA) was then carried out to visualize the global variation in the observations and to detect possible outliers. The quality controls, which are the mix of all samples, were located in the middle of the PCA scatter plot demonstrating the reliable performance and reproducibility of the LC-MS analysis (Supplementary Fig. 1). None of the observations had to be removed as outliers. Supervised classification was performed using the orthogonal projections to latent structures discriminant analysis (OPLS-DA) model. In addition to the classic PLS model, the orthogonal PLS model separates the dataset variation into two parts: predictive and orthogonal, and therefore allows improved interpretability^29,30^. The optimum OPLS-DA model for RCC histological subtype classification was established with 2 predictive components and 1 orthogonal component, a R^2^X_(cum)_ of 44.2% and a goodness-of-fit R^2^ of 75.0% (Fig. 1a). The model shows discrimination between all three main subtypes of RCC. Moreover, all 37 samples were classified correctly according to their corresponding subtype.

Cross-validation tests and permutation tests were performed to evaluate the predictive capability of this model. For cross-validation tests we withheld, by turns, 1/7 of the cohort as validation sets. The goodness-of-prediction Q^2^ estimated from these validation sets was 59.7%. The p-value of cross-validation ANOVA test at 7.516 × 10^−8^ also showed the strong predictive power of the model. The permutation tests randomly transformed the class list of the cohort and compared the newly created models to the existing ones. All 100 permutation models presented lower predictive power Q^2^ than the initial model. The Q^2^-intercept was −0.4 (Fig. 1b). Both tests showed that this RCC subtype classification is statistically validated and of significant predictive power.

In order to evaluate the prognostic performance of the OPLS-DA model for RCC subtype classification, 28 additional samples (19 clear cell, 5 papillary and 4 chromophobe) were collected as the validation set and analysed in the same manner as the training set (Tables 1 and 2). The RCC subtype determined by histology, and those predicted by the OPLS-DA model, were used to calculate the ROC curve. The area under curve (AUC) and 95% confidence interval was 0.924 (0.799–1.049), 0.991 (0.964–1.018), 1 (1–1), respectively, for ccRCC, papRCC and chroRCC (Fig. 1c). These results showed that, depending on the tumour subtype, at least 92% of the samples could be diagnosed accurately.

To better understand the most discriminative metabolites for RCC subtype classification, the weight of each metabolite on each component is visualized on a loading plot (Fig. 1d). Metabolites that contribute more to the discrimination will have a higher weight, and thus, tend to be the farthest away from the origin. The importance of each metabolite was summarized by the variable influence on projection (VIP) (Supplementary Table 1). For all metabolites with a VIP >3 in subtype classification, we compared their variation in each subtype and in healthy controls (Table 3).

**Table 3:** Levels of the most discriminative metabolites (VIP >3) for RCC subtype classification.

Name	m/z	Mode	VIP	Peak intensity			Ratio			Peak Intensity	Ratio
				Mean cc	Mean pap	Mean chro	pap/cc	chro/cc	pap/chro	Mean Healthy	RCC/Healthy
Creatine	132.07694	POS	10.51	2.98E + 09	1.31E + 09	4.92E + 09	0.44	1.65	0.27*	2.06E + 09	1.26
L-Acetylcarnitine	204.12320	POS	10.43	4.50E + 09	1.91E + 09	3.13E + 09	0.42**	0.7	0.61	2.90E + 09	1.24
L-Carnitine	162.11262	POS	6.85	2.18E + 09	1.03E + 09	1.79E + 09	0.47*	0.82	0.58	1.34E + 09	1.82
L-Proline	116.07095	POS	6.45	1.39E + 09	2.06E + 09	8.66E + 08	1.48*	0.62	2.38***	1.56E + 09	0.71*
L-Phenylalanine	166.08636	POS	6.07	1.44E + 09	2.07E + 09	1.09E + 09	1.43	0.76	1.90*	2.09E + 09	0.51*
Betaine	118.08658	POS	5.15	2.10E + 09	2.46E + 09	1.82E + 09	1.17	0.87	1.35	1.71E + 09	1.44
Hypoxanthine	137.04591	POS	5.11	1.21E + 09	1.95E + 09	1.79E + 09	1.61*	1.48	1.09	3.26E + 09	0.54**
L-Lactic acid	89.02295	NEG	5	3.45E + 09	3.87E + 09	3.55E + 09	1.12	1.03	1.09	2.16E + 06	0.8
L-Isoleucine	132.10212	POS	4.65	1.44E + 09	1.51E + 09	8.21E + 08	1.05	0.57	1.83*	1.26E + 09	0.56**
Glycerophosphocholine	296.06608	POS	4.03	4.80E + 08	5.79E + 08	8.33E + 08	1.21	1.73	0.7	2.86E + 08	2.06
L-Methionine	150.05848	POS	3.89	2.18E + 08	6.83E + 08	2.83E + 08	3.14**	1.3	2.41*	6.15E + 08	0.36**
L-Tyrosine	182.08135	POS	3.7	3.99E + 08	7.36E + 08	3.26E + 08	1.85**	0.82	2.26***	9.71E + 08	0.49*
L-Palmitoylcarnitine	400.34272	POS	3.66	4.48E + 08	1.05E + 08	1.84E + 08	0.23**	0.41	0.57	1.17E + 08	0.96
Dimethylglycine	104.07109	POS	3.63	4.50E + 08	3.42E + 08	3.94E + 07	0.76	0.09***	8.67*	1.28E + 08	1.01
L-Tryptophan	205.09736	POS	3.4	4.34E + 08	6.37E + 08	2.85E + 08	1.47	0.66	2.24*	7.77E + 08	0.41**
Hydroxybutyrylcarnitine	248.14922	POS	3.22	4.08E + 08	1.22E + 08	5.12E + 08	0.3	1.26	0.24	2.50E + 08	2.62*
N-Acetyl-L-aspartic acid	174.03976	NEG	3.21	3.81E + 07	8.15E + 07	3.57E + 08	2.14	9.37**	0.23*	5.97E + 08	0.29*
Adenosine	268.10419	POS	3.19	1.19E + 08	1.09E + 08	4.88E + 08	0.91	4.1	0.22	1.45E + 08	1.37
L-Malic acid	133.01299	NEG	3.19	4.93E + 08	2.53E + 08	2.23E + 08	0.51	0.45*	1.14	5.19E + 08	0.63
Isobutyryl-L-carnitine	232.15447	POS	3.14	4.66E + 08	3.39E + 08	6.25E + 08	0.73	1.34	0.54	4.82E + 08	1.39
L-Glutamic acid	148.06055	POS	3.07	1.09E + 09	7.98E + 08	5.56E + 08	0.73	0.51	1.43	7.57E + 08	1.26

p-values were calculated using a Mann-Whitney test for unpaired comparison and a Wilcoxon signed rank test for paired comparison. The level of significance was set at *for p < 0.05, **for p < 0.01 and ***for p < 0.001.

In addition, an OPLS-DA model was constructed for the discrimination of RCC samples from paired normal tissues (Supplementary Fig. 2). The fitting and predictive parameters of the model are R^2^X_(cum)_ of 55.5%, R^2^ of 93.2%, Q^2^ of 78.0%, CV-ANOVA p-value of 0.015 and Q^2^-intercept of the permutation test of −0.5. Our dataset demonstrated that the model is statistically valid despite the limited sample number.

### Metabolic pathway analysis

**Figure 2:**
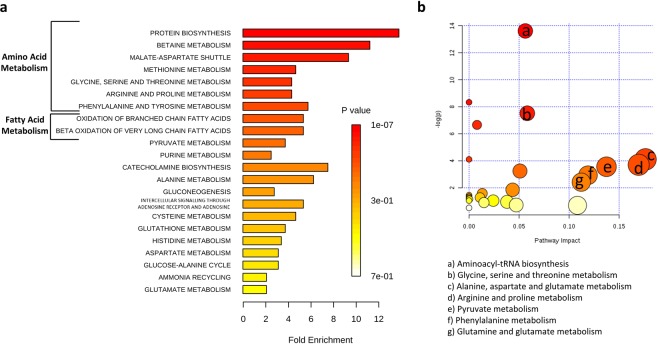
Pathway analysis of altered metabolites in RCC subtypes. (**a**) Metabolite set enrichment analysis using SMPDB (Small Molecule Pathway Database). (**b**) Metabolomic pathway analysis using the KEGG database.

For a better understanding of metabolic dysregulation among RCC subtypes, we performed two types of pathway analysis. Metabolite Set Enrichment Analysis was performed using the Small Molecule Pathway Database (Fig. 2a) and Metabolic Pathway Analysis was performed using the KEGG database, which also calculates the impact of each pathway using topology analysis in addition to the classic enrichment analysis^31,32^ (Fig. 2b). Amino acid metabolism appeared to be the most frequently modified pathways in both analyses. These included methionine, arginine and proline, phenylalanine, glycine, serine and threonine metabolism, among others. Enrichment and pathway analyses also showed modifications in fatty acid and pyruvate metabolism.

### Heterogeneity within a tumour

**Figure 3:**
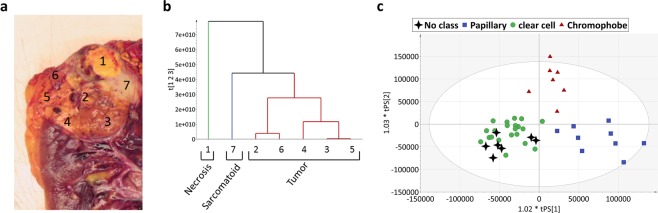
Example of metabolic profiling in tumour heterogeneity. (**a**) Macroscopic observation. (**b**) Metabolic profiling. Dendrogram of hierarchical clustering analysis (HCA). (**c**) Predicted scores of the 7 in-tumour heterogeneity samples in the OPLS-DA RCC subtype classification model. All 7 samples (black stars) were correctly predicted according to their subtype, ccRCC.

For 6 ccRCC, several samples were collected from each surgical piece to evaluate the heterogeneity within the tumours using metabolomics. We performed a hierarchical cluster analysis (HCA) on each set of data. For 1 of the 6 tumours, within the same surgical specimen, we observed a necrosis zone, a sarcomatoid zone and a conventional ccRCC zone. The HCA classification clearly distinguishes the necrosis portion and the sarcomatoid portion from the conventional carcinoma portion. This classification correlates with the results observed from macro and micro analyses (Fig. 3a,b). Interestingly, when we integrated all these samples in our previous OPLS-DA RCC subtype classification model, despite the intra-tumour heterogeneity, all samples were correctly predicted to be clear cell subtype (Fig. 3c). It is important to note that we were also able to correctly classify all samples from the other 5 ccRCC tumours (data not shown).

**Figure 4:**
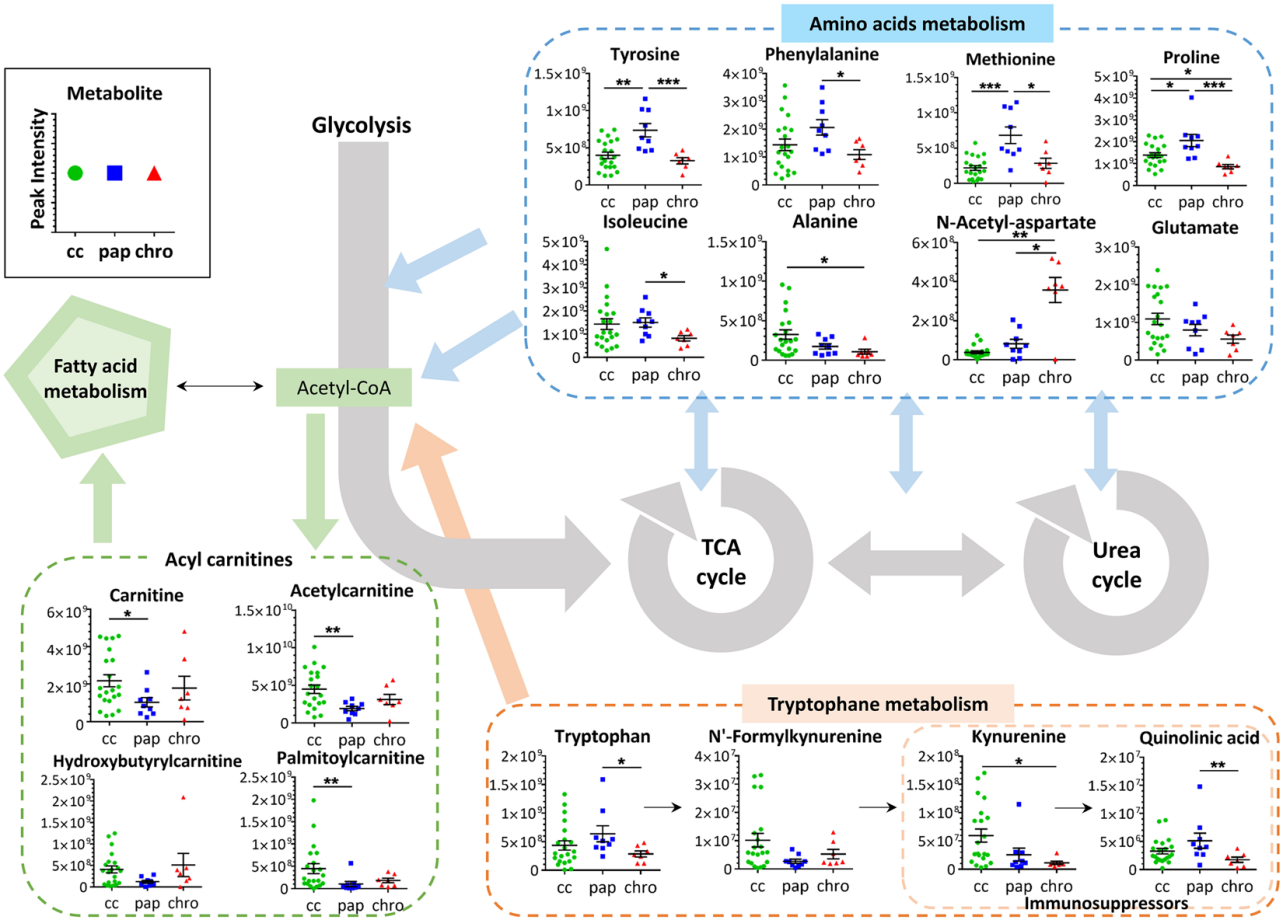
Main metabolic dysregulation among clear cell (cc), papillary (pap) and chromophobe (chro) RCC. Data were shown as the mean ± SEM. p-values were calculated using a Mann-Whitney test. The level of significance was set at *for p < 0.05, **for p < 0.01 and ***for p < 0.001.

## Discussion

In this work, we show for the first time that untargeted metabolomics allows the classification of all three main RCC histologic subtypes. In addition, our results provide new insights into the molecular characteristics of each subtype.

Precise classification of RCC subtypes has important implications in kidney cancer treatment. It provides precious information, not only on the clinical tumour behaviour, but also on cancer prognosis and it could also help direct therapeutic strategies^8^. Furthermore, up to 5% of RCC cannot be classified using standard histologic observations^33^, therefore the development of complementary methods for RCC subtype classification is of critical importance to ensure the accuracy and robustness of cancer diagnoses. Recent technological progress with respect to the speed and resolution of LC-MS metabolomic analyses makes this approach particularly promising. Here, we present the first study showing the feasibility of the classification of all three main RCC subtypes using metabolomic profiling. Combining LC-MS with multivariate analyses, we were able to build an OPLS-DA model with a fit (R^2^) of 75.0% and a prediction (Q^2^) of 59.7%. The predictive power of the model was validated by cross validation tests (CV ANOVA p-value = 7.516 × 10^−8^) and permutation tests (Q^2^-intercept = −0.4). The high prognostic performance of the model was also demonstrated by an independent sample cohort using ROC analyses showing that, depending on RCC subtype, at least 92% of samples were accurately predicted using metabolomic data. Thus, we have demonstrated that metabolic profiling could provide a new tool for RCC subtype diagnosis. Our study also indicates that limited tissue sample sizes (10–20 mm^3^) should be sufficient according to the LC-MS/MS analyses that were performed on a small aliquot of the extracted metabolites. In addition, most discriminative metabolites were detected at an intensity 10^2^ to 10^4^-fold higher than the noise level. Therefore, such analyses should work on more clinically accessible samples, as renal biopsies. Compared to commonly used analyses (histologic, immunohistologic and genetic)^34^, metabolomic analyses give information about the molecular features of the tumour, and therefore provide additional information on tumour behaviour. Moreover, it presents the advantages of being fast, cheap and robust.

The second aim of this study was to elucidate differences between RCC subtypes at a molecular level. To date, the vast majority of clinical trials and drug development strategies have focused on clear cell RCC due to its high frequency (over 75% of RCCs). Subsequent therapeutic strategies are then similar for the treatment of the other subtypes. However, it is known that renal cell carcinoma is a very heterogeneous group of tumours that display different behaviours. The RCC subtypes differ in their histologic appearance, genetic profile and response to drug treatment. To develop new subtype-specific therapeutic approaches, it is essential to understand the differences and similarities between subtypes on a molecular level^11^. In this study we have established an extensive overview of the metabolic profiles of the main RCC subtypes. Over 1000 metabolites were identified and semi-quantified for each RCC subtype. Our data suggest that metabolic reprogramming mechanisms are different between RCC subtypes. Moreover, pathway analyses revealed significant differences between the RCC subtypes, mainly concerning amino acid and fatty acid metabolism.

As previously shown by both Hakimi and Ganti and their collaborators^16,35^, we found that the levels of numerous amino acids (tyrosine, tryptophan, isoleucine, methionine, proline, and phenylalanine) were decreased in RCC samples compared to control samples. Our results reveal that the levels of different classes of amino acids were differentially altered in each RCC subtype (Fig. 4). Consistent with the study of Schaeffeler and collaborators^26^, we observed that multiple amino acid metabolic pathways were modified between ccRCC and chroRCC, such as glycine, serine and threonine metabolism and methionine metabolism. We also found that several amino acids or their derivatives, for example N-acetyl-L-aspartate, glutamate and alanine, had more similarities between chroRCC and healthy control samples compared to the other two subtypes. This aligns with a previous study^26^ which showed that the metabolic coregulation network is more altered in ccRCC than chroRCC compared to non-tumorous tissue. Interestingly, the levels of all three listed amino acid-associated metabolites varied in a progressive way with the chroRCC being the most similar to the healthy sample, then the papRCC, with ccRCC being the most divergent. To the contrary, the level of aromatic amino acids, such as tryptophan, tyrosine and phenylalanine, were significantly higher in papRCC compared to ccRCC and chroRCC. A similar profile was observed for methionine and proline. The dysregulation of amino acid metabolism is known to be a key event during cancer development^36^ and alterations in specific amino acid levels are emerging hallmarks of cancers. Amino acids serve, not only as basic building blocks in protein synthesis, but also as metabolic regulators in cancer cell growth^37^. Our results suggest that the different RCC histologic subtypes are each using very specific amino acids as energetic sources for cell proliferation.

Pathway analysis also revealed alterations in fatty acid metabolism. Several studies^25–27,35^ have found that ccRCC samples have increased fatty acid metabolism compared to healthy samples, which was not the case for chroRCC. Accordingly, our results showed that, compared to normal tissues, the level of acylcarnitines, such as carnitines, palmitoylcarnitines and acetylcarnitines, are only increased in ccRCC, whereas the papillary and chromophobe subtypes have a similar level of acylcarnitines as in normal tissues. It has previously been reported by Ganti and collaborators^21^ that acylcarnitine concentrations are increased in the urine of ccRCC patients. It is also known that only clear cell subtypes accumulate excessive intracellular lipid and glycogen, which accounts for their clear appearance in histological observations^38^. Thus, the elevated level of acylcarnitines in ccRCC relative to the other two subtypes is consistent with its increased fatty acid metabolism.

Another interesting difference is the variation in the levels of immune-suppressive metabolites in the tryptophan pathway. As shown in Fig. 4, increases in kynurenine levels were frequently observed in ccRCC samples, whereas the majority of papRCC and chroRCC showed low kynurenine levels. Based on these observations, we could hypothesize that therapies targeting the kynurenine immune-suppressive effect (i.e. the indoleamine 2,3-dioxygenase (IDO) inhibitor but also interferon-α-based, interlekin-2-based and more recently anti-PD1 based immunotherapies)^10,39^ may have a lower success rate in papRCC and chorRCC than in ccRCC. However, further research with a much higher number of patients is required to clearly demonstrate the efficiency of metabolomic analyses in the prediction of successful cancer treatment strategies.

It has to be noted that despite the significant differences in metabolite levels between the RCC subtypes, we could not identify any individual metabolite that independently allowed for correct and reliable classification. We show that the integration of complex metabolic profiling using multivariate analyses provides a more accurate and robust tool than the use of only one or several metabolites as a biomarker.

Finally, this study revealed that metabolomics also allows the characterization of different zones within a single tumour (necrosis, carcinoma or sarcomatoid). Importantly, this heterogeneity does not interfere with the overall subtype ccRCC classification, thus confirming the reliability of this metabolomics approach for subtype classification.

To conclude, this study demonstrated that all three main RCC subtypes can be discriminated using an untargeted metabolomics approach. Furthermore, we detected significant differences in metabolic profiles (amino acid and fatty acid metabolism) among RCC subtypes, which should be useful for the development of new specific treatments of papRCC and chroRCC. Our findings highlight the importance of metabolomic analyses, not only for RCC subtype classification, but also for elucidating the behaviour of different RCC subtypes.

## Methods

### Sample collection and histological subtype diagnosis

Tissue samples from 61 patients with non-metastatic RCC that had undergone surgery in the urology department of the Nice University Hospital between May 2016 and May 2018 were selected. As defined by the 2016 World Health Organization criteria^40,41^, diagnosis was based upon pathology and cytogenetic analysis. Initial management of surgical specimens was performed according to a standardized protocol. The surgical specimens were obtained immediately after nephrectomy. Fresh samples were collected and frozen in liquid nitrogen for further metabolomic analyses. We confirmed the identity of the examined tissue by microscopic examination of a mirror formalin-fixed paraffin-embedded (FFPE) sample. 51 patients having a tumour defined as ccRCC, papRCC or chroRCC were enrolled. The cohort was secondarily completed by including 14 patients with archived frozen tissue to complete the number in non ccRCC subtypes. All the tumours were managed in the same way, for both the initial macroscopic procedure and the diagnosis procedure. The detailed clinical pathological parameters for all the patients included in this study and their grade distribution are reported in Tables 1 and 2. Informed consent was obtained from all individual participants included in the study. The study included only the adult patients. All of the samples are the property of the tissue collection of the Pathology Department of the University Hospital of Nice and are declared annually to the French Health Ministry. The procedures followed were approved by the institutional review board of the University Hospital of Nice. This study was conducted in accordance with the Declaration of Helsinki.

### Sample preparation

Frozen tissues (~200 mm^3^) were placed in microcentrifuge tubes and ground in 1 mL cold methanol (LC-MS grade, Merck Millipore, Molsheim, France) using pestles. Homogenized samples were incubated overnight at −20 °C then centrifuged at 15 000 g for 15 minutes. The supernatants were removed and dried using a SpeedVac concentrator (SVC100H, SAVANT, Thermo Fisher Scientific, Villebon-sur-Yvette, France). The lyophilized samples were resuspended in 180 µL of 50:50 acetonitrile-H_2_O mix (LC-MS grade, Merck Millipore) prior to LC-MS/MS analyses.

### LC-MS/MS analysis

Metabolic profiling was performed using LC-MS/MS. Liquid chromatographic analysis was performed using the DIONEX Ultimate 3000 HPLC system (Thermo Fisher Scientific). A 10 µL of each sample was injected onto a Synergi 4 µm Hydro-RP 80 Å, 250 × 3.0 mm column (Phenomenex, Le Pecq, France). The mobile phases were composed of 0.1% formic acid (Thermo Fisher Scientific) in water (A) and 0.1% formic acid in acetonitrile (B). The gradient was set as follows with a flow rate of 0.9 mL/min: 0% phase B from 0 to 5 min, 0–95% B from 5 to 21 min, holding at 95% B to 21.5 min, 95–0% B from 21.5 to 22 min, holding at 0% B until 25 min for column equilibration. Mass spectrometry analysis was carried out on a Q Exactive Plus Orbitrap mass spectrometer (Thermo Scientific) with a heated electrospray ionization source, HESI II, operating in both positive and negative mode. High-resolution accurate-mass full-scan MS and top 5 MS2 spectra were collected in a data-dependent fashion at a resolving power of 70 000 and 35 000 at m/z 400, respectively. A quality control (QC) sample was prepared from an equal mix of all collected samples. It was injected at the beginning of the run and after every 9 samples in order to monitor the stability of the mass spectrometer performance.

### Metabolomic profiling

Raw data files were converted to mzXML files using MSConvert (Version 2.1, ProteoWizard)^42^. The data obtained from positive and negative ionization mode were analysed separately using MZmine (Version 2.29)^43^. Isolated chromatograms were built for each mass with a noise threshold of 10^5^. A local minimum search algorithm was used to select the validated peaks. Peaks were then aligned by RANSAC (random sample consensus) algorithm with a tolerance of 10 ppm in m/z and 1 min in retention time. Missing values were filled in using the same m/z and RT range as observed in detected samples, where possible. We kept only those peaks that had no missing values after gap-filling. Peaks were then identified using the Human metabolome database^44^ (HMDB, version 3.0) with 15 ppm of mass tolerance. All selected metabolites with VIP >3 were individually verified (MS and MS2 spectra). Only identified metabolites were kept for further analyses. Results obtained with each polarity were combined, and for metabolites that were identified in both modes, we kept the metabolites with higher intensity mean.

### Multivariate analysis

For multivariate analysis, metabolomic data were introduced into the SIMCA software (Version 14.1, Sartorius Stedim Biotech, Germany). Raw data were mean-centred and scaled with the square root of the standard deviation (Pareto scaling)^45^. At first, principal component analysis (PCA) was carried out using scaled data to visualize the overview of the dataset^46^. Outliers were eliminated if observed. Orthogonal projections to latent structures discriminant analysis (OPLS-DA), a supervised model, was then established to relate the X data to the Y response^29,30^. In our case, the X is the metabolomic dataset and the Y is the histological classes allowing for the discrimination of different RCC subtypes. The OPLS-DA model was evaluated using R^2^X_(cum)_: the variation of X explained by the model; R^2^: the goodness-of-fit that is represented by the percentage of the variation of Y explained by the model; Q^2^: the goodness-of-prediction. Q^2^ of the model was evaluated using a cross-validation test. 1/7 of the data were withheld during model development. The withheld portion was then predicted by the model that had been established using the remaining 6/7 of the data. The predictions for the excluded parts were compared with the actual values and these steps were repeated until all data had been withheld once. Q^2^ is the percentage of the variation of the dataset predicted by the model according to the cross-validation test. The significance of the cross-validation test was calculated by ANOVA using the predictive residuals. A significant model should have a CV-ANOVA p-value of less than 0.05. Additionally, response permutation tests were performed for the model validation. To this end, the Y (classes) are permutated to appear in a different order while the X-dataset remains intact. A new model is fitted to the permutated data. The R^2^ and Q^2^ of the permutated model are compared to the real model. A validated model should have all permutated Q^2^ values lower than the original Q^2^ and the Q^2^-intercept below zero.

The influence of each metabolite on the classification was calculated by the variable influence on projection (VIP)^30^. Metabolites with VIP >1 have an above average influence. In our case, only metabolites with VIP values >3 were selected for further pathway analysis using free web-based software, MetaboAnalyst (Version 3.0)^32^. Additionally, annotation of metabolites with VIP >3 was verified manually using a reference MS/MS spectrum of the Metlin database. About 15% of identifiers were invalidated, mainly due to the confusion between isomers and the presence of contaminants from the mass spectrometer.

### Statistical analyses

Univariate analyses were carried out using GraphPad Prism (Version 5.03, GraphPad Software Inc., USA). Differences between unpaired groups were compared using the two-tailed Mann-Whitney test. For paired data, the two-tailed Wilcoxon matched-pairs signed rank test was applied. Data were shown as the mean ± SEM. The level of significance was indicated by *for p < 0.05, **for p < 0.01 and ***for p < 0.001. Hierarchical cluster analyses (HCA) were performed by SIMCA software using Ward’s method^47^. The receiver operating characteristic (ROC)^48^ curves were calculated using GraphPad Prism. The prognostic performance of the metabolomic-based OPLS-DA model was evaluated using an independent sample cohort by computing the area under the ROC curve (AUROC) using the predicted Y values for each RCC subtype.

## Supplementary information

Supplementary Information

Supplementary Dataset
