# Supplementary material for: LC-MS based metabolomic profiling for renal cell carcinoma histologic subtypes
Source: Sci Rep. 2019 Oct 30;9:15635. doi: 10.1038/s41598-019-52059-y (PMC6821699; doi:10.1038/s41598-019-52059-y)
Supplement: Supplementary file 1 — Supplementary Information [file 41598_2019_52059_MOESM1_ESM.pdf]

LC-MS based metabolomic profiling for renal cell carcinoma histologic subtypes

Lun Jing, Jean-Marie Guigonis, Delphine Borchiellini, Matthieu Durand, Thierry Pourcher, and Damien Ambrosetti

Supplementary information

Supplementary Figure 1

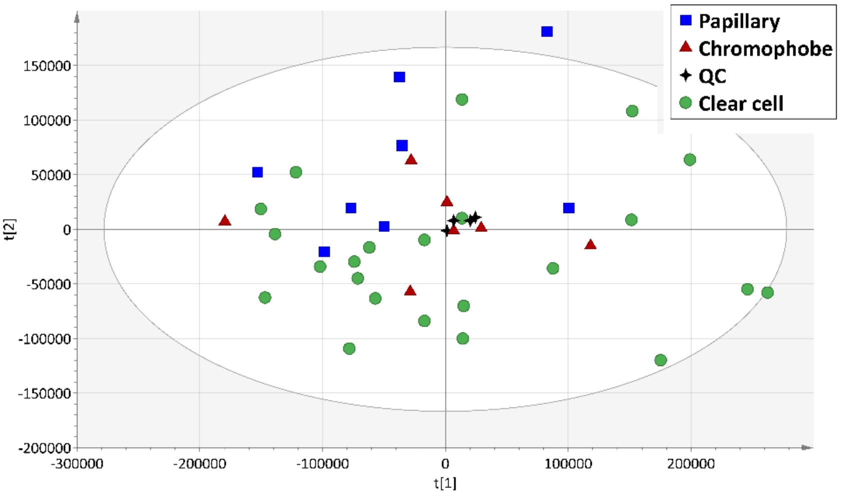

Supplementary Figure 1. Principal component analysis (PCA) of RCC subtype based on untargeted metabolomics data.

Supplementary Figure 2

A

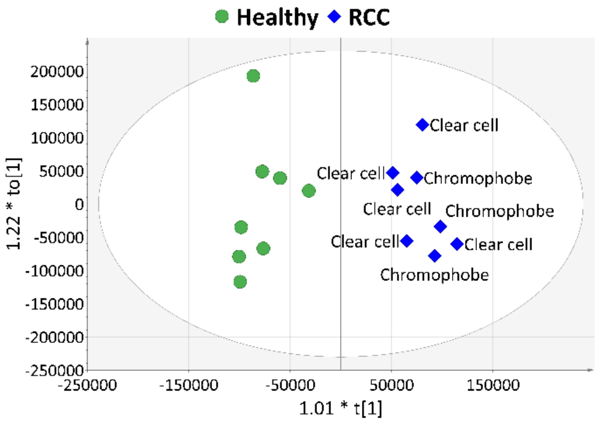

B

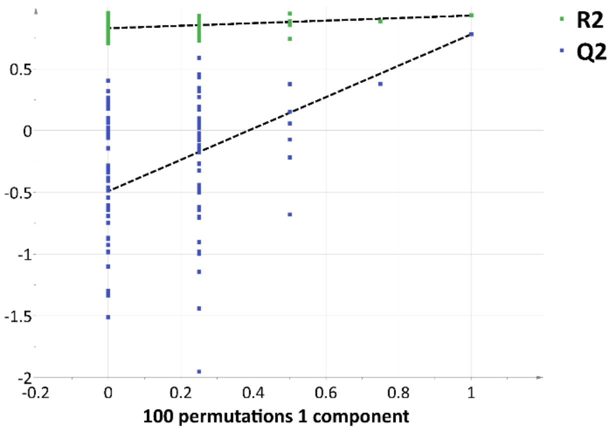

C

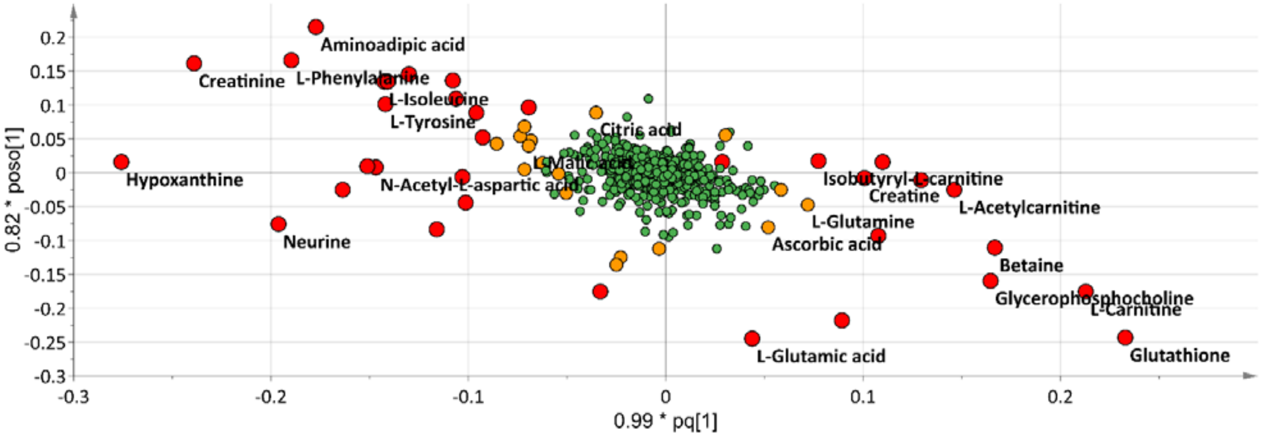

Supplementary Figure 2. Discrimination between healthy controls and renal cell carcinomas based on untargeted metabolomics data. A. OPLS-DA analysis showing the discrimination between healthy control and carcinoma samples (8 matched pairs including 5 clear cell and 3 chromophobe). The model presents 1 predictive component, 2 orthogonal components, $R^2X_{(cum)}$ of 55.5%, a goodness-of-fit R^2 of 93.2%, a goodness-of-prediction Q^2 of 78.0% and CV-ANOVA p-value of 0.015. **B. Validation plot obtained from 100 permutation tests. C. Loading plot showing the most discriminative metabolites in normal and RCC tissue discrimination.** The metabolites with VIP (Variable Importance for the Projection) > 3 are highlighted with red circles; with VIP > 2 are highlighted with orange circles.

Supplementary Table 1. Variable influence on projection (VIP) for RCC subtype classification.

Var ID (Primary)	Var ID (row m/z)	Var ID (ID)	M3.VIP[2+1+0]
**Creatine**	132.077	HMDB00064	10,5111
**L-Acetylcarnitine**	204.123	HMDB00201	10,4271
**L-Carnitine**	162.113	HMDB00062	6,84946
**L-Proline**	116.071	HMDB00162	6,45495
**L-Phenylalanine**	166.086	HMDB00159	6,07172
**Dihydrothymine**	129.066	HMDB00079	5,16308
**Betaine**	118.087	HMDB00043	5,1483
**Hypoxanthine**	137.046	HMDB00157	5,10661
**L-Lactic acid**	89.0229	HMDB00190	4,99924
**L-Isoleucine**	132.102	HMDB00172	4,65421
**5-Aminoimidazole ribonucleotide**	296.066	HMDB01235	4,03263
**L-Methionine**	150.058	HMDB00696	3,89251
**Humulinic acid A**	265.148	HMDB30104	3,77026
**L-Tyrosine**	182.081	HMDB00158	3,69869
**L-Palmitoylcarnitine**	400.343	HMDB00222	3,6649
**Dimethylglycine**	104.071	HMDB00092	3,62773
**L-Tryptophan**	205.097	HMDB00929	3,40488
**Hydroxybutyrylcarnitine**	248.149	HMDB13127	3,2158
**N-Acetyl-L-aspartic acid**	174.04	HMDB00812	3,2133
**Adenosine**	268.104	HMDB00050	3,19064
**L-Malic acid**	133.013	HMDB00156	3,18659
**Isobutyryl-L-carnitine**	232.154	HMDB00736	3,14472
**L-Glutamic acid**	148.061	HMDB00148	3,0703
**Glutathione**	308.091	HMDB00125	2,9841
**Glycerophosphocholine**	258.11	HMDB00086	2,96531
**trans-Hexadec-2-enoyl carnitine**	398.327	HMDB06317	2,94701
**Tetradecanoylcarnitine**	372.311	HMDB05066	2,89112
**Nalbuphine**	358.201	HMDB14982	2,82291
**Beta-Alanine**	90.0555	HMDB00056	2,76142
**Oleoylcarnitine**	426.358	HMDB05065	2,64926
**Neurine**	104.107	HMDB31259	2,56708
**Linoelaidyl carnitine**	424.342	HMDB06461	2,50077
**Aminoadipic acid**	162.076	HMDB00510	2,47727
**Methylmalonic acid**	117.018	HMDB00202	2,42256
**Isoplumbagin**	187.041	HMDB35291	2,40383
**N-Desmethylvenlafaxine**	264.196	HMDB13892	2,37449
**Cysteineglutathione disulfide**	214.051	HMDB00656	2,36515
**Guanidoacetic acid**	118.061	HMDB00128	2,28672
**Indoleacrylic acid**	188.071	HMDB00734	2,27785
**Adenine**	136.062	HMDB00034	2,24758
**2-Octenoylcarnitine**	286.201	HMDB13324	2,24301
**Adenosine monophosphate**	348.07	HMDB00045	2,23197
**LysoPC(16:0)**	496.34	HMDB10382	2,19013
**Pyrrolidine**	72.0814	HMDB31641	2,13555
**Hexanoylcarnitine**	260.186	HMDB00705	2,06003
**N-a-Acetyl-L-arginine**	217.129	HMDB04620	2,03322
**L-1,2,3,4-Tetrahydro-beta-carboline-3-carboxylic acid**	217.097	HMDB35665	1,97511
**Acetaminophen**	152.071	HMDB01859	1,95179
**Citramalic acid**	147.029	HMDB00426	1,92319
**cis-5-Tetradecenoylcarnitine**	370.295	HMDB02014	1,88553
**Tyrosol 4-sulfate**	110.02	HMDB41785	1,85842
**N-Methylethanolaminium phosphate**	156.042	HMDB60173	1,85049
**Niacinamide**	123.056	HMDB01406	1,83226
**Valganciclovir**	353.161	HMDB15548	1,80062

**3-Hydroxy-11Z-octadecenoylcarnitine**	442.353	HMDB13339	1,79403
**Galactonic acid**	195.05	HMDB00565	1,74207
**Spermidine**	146.165	HMDB01257	1,71571
**Imidazole acetol-phosphate**	111.021	HMDB12236	1,70828
**Alnustone**	263.146	HMDB31664	1,67864
**Xanthine**	151.025	HMDB00292	1,65658
**Pyridine N-oxide glucuronide**	273.084	HMDB61177	1,65468
**PC-M6**	420.251	HMDB38568	1,64902
**3-Methyl sulfolene**	133.032	HMDB59667	1,64129
**p-Cresol sulfate**	187.006	HMDB11635	1,63924
**N6-Acetyl-L-lysine**	189.124	HMDB00206	1,61829
**Methoxsalen**	215.032	HMDB14693	1,61131
**Lidocaine**	235.181	HMDB14426	1,59592
**Dihydrouracil**	115.051	HMDB00076	1,59318
**Proline betaine**	144.102	HMDB04827	1,59157
**Taurine**	124.006	HMDB00251	1,58823
**L-Lysine**	147.113	HMDB00182	1,57718
**Dichloromethane**	84.9603	HMDB31548	1,56566
**Dodecanoylcarnitine**	344.279	HMDB02250	1,53279
**2-O-Galloyl-1,4-galactarolactone**	343.026	HMDB37199	1,51337
**N-Methylnicotinamide**	137.071	HMDB03152	1,46751
**Triethanolamine**	150.113	HMDB32538	1,43795
**N-Heptanoylglycine**	188.128	HMDB13010	1,43557
**Uric acid**	167.02	HMDB00289	1,43158
**3-Hydroxyhexadecanoylcarnitine**	416.337	HMDB13336	1,43105
**Piperidine**	86.097	HMDB34301	1,42661
**Glycerylphosphorylethanolamine**	214.048	HMDB00114	1,41495
**PC(14:0/20:1(11Z))**	760.584	HMDB07879	1,41027
**Ribose-1-arsenate**	272.959	HMDB12285	1,40237
**3(S)-hydroxy-13-cis-docosenoyl-CoA**	130.017	HMDB62200	1,39011
**DL-2-Aminooctanoic acid**	160.133	HMDB00991	1,37792
**hydroxyisovaleroyl carnitine**	262.165	HMDB62555	1,3641
**Isoleucyl-Isoleucine**	245.186	HMDB28910	1,35068
**LysoPE(0:0/18:1(11Z))**	480.309	HMDB11475	1,34772
**Diphenyl disulfide**	219.027	HMDB31823	1,34432
**Dehydroxyzyleuton**	111.039	HMDB13970	1,32777
**Isoleucyl-Valine**	231.17	HMDB28920	1,32402
**Phenylpyruvic acid**	165.055	HMDB00205	1,29824
**Ascorbic acid**	175.024	HMDB00044	1,28794
**3,5,3-triiodothyronine-4-sulfate**	129.02	HMDB62205	1,28414
**5'-Methylthioadenosine**	298.097	HMDB01173	1,27028
**Amoxapine**	312.095	HMDB14683	1,27022
**Diisopropyl sulfide**	119.09	HMDB29579	1,26252
**Selenium Sulfide**	112.896	HMDB15106	1,25687
**N2-Succinyl-L-ornithine**	233.113	HMDB01199	1,22232
**L-Kynurenine**	209.092	HMDB00684	1,21881
**N-Acetylarylamine**	136.076	HMDB01250	1,19761
**1-Oxo-1H-2-benzopyran-3-carboxaldehyde**	88.0237	HMDB30577	1,19331
**1-Pyrroline**	70.0658	HMDB12497	1,19245
**Hippuric acid**	178.05	HMDB00714	1,18142
**Dibenzyl disulfide**	245.043	HMDB32077	1,15664
**3-hydroxyoctanoyl carnitine**	304.212	HMDB61634	1,1535
**(2S,4R)-4-(9H-Pyrido[3,4-b]indol-1-yl)-1,2,4-butanetriol**	273.121	HMDB35191	1,13778
**1-(Isothiocyanatomethyl)-4-methoxybenzene**	90.5264	HMDB32581	1,12798
**Trigonelline**	138.055	HMDB00875	1,1251
**Ophthalmic acid**	290.135	HMDB05765	1,12354
**Gamma-Butyrolactone**	87.0446	HMDB00549	1,10032
**Thiolutin**	226.996	HMDB34228	1,09423
**5-Hydroxyisourate**	185.032	HMDB30097	1,09399

**9,12-Hexadecadienoylcarnitine**	396.311	HMDB13334	1,09208
**Lenticin**	247.144	HMDB61115	1,08828
**Aniline**	94.0656	HMDB03012	1,08206
**L-argininium(1+)**	176.128	HMDB62762	1,08044
**Ethyl aconitate**	203.053	HMDB40275	1,07958
**1-(4-Hydroxy-3-methoxyphenyl)-3-decanone**	279.192	HMDB30801	1,07098
**LysoPC(18:1(9Z))**	522.355	HMDB02815	1,06414
**Inosine**	267.073	HMDB00195	1,06232
**2-Hydroxyadipic acid**	161.044	HMDB00321	1,0502
**Ureidopropionic acid**	133.061	HMDB00026	1,04489
**Pseudoecgonine**	186.113	HMDB06348	1,03703
**3-Hydroxy-2-methyl-[R-(R,S)]-butanoic acid**	117.054	HMDB00351	1,03695
**Phosphodimethylethanolamine**	168.042	HMDB60244	1,02397
**3,4-Dihydroxymandelic acid**	185.042	HMDB01866	1,02144
**Caffeine**	195.088	HMDB01847	1,01403
**3-Hydroxy-9-hexadecenoylcarnitine**	414.321	HMDB13333	1,01221
**Valerenic acid**	235.166	HMDB30016	1,0086
**Creatinine**	114.067	HMDB00562	1,00418
**Uridine 5'-monophosphate**	323.029	HMDB00288	0,999177
**N-(4-hydroxyphenyl)ethoxycarbothioamide**	99.5316	HMDB33179	0,966969
**2,5-Dichloro-4-oxohex-2-enedioate**	226.952	HMDB60363	0,955923
**3-Hexenedioic acid**	145.05	HMDB00393	0,945752
**Propionylcarnitine**	218.139	HMDB00824	0,939206
**2-Methyl-3-ketovaleric acid**	129.054	HMDB00408	0,937506
**Pseudoephedrine**	166.123	HMDB01943	0,933523
**2,5-Furandicarboxylic acid**	157.011	HMDB04812	0,929247
**Salsoline-1-carboxylate**	236.092	HMDB13067	0,927692
**L-Arginine**	175.119	HMDB00517	0,900684
**3-hydroxydecanoyl carnitine**	332.243	HMDB61636	0,89175
**Phosphoric acid**	96.9682	HMDB02142	0,888322
**Fenamiphos**	302.101	HMDB31787	0,863479
**Spermine**	203.223	HMDB01256	0,85868
**2-Hydroxylauroylcarnitine**	360.275	HMDB13164	0,858169
**Gravelliferone**	297.153	HMDB30729	0,845069
**L-Glutamine**	147.077	HMDB00641	0,842453
**L-Octanoylcarnitine**	288.217	HMDB00791	0,842017
**Cytidine**	242.08	HMDB00089	0,836793
**Erythronic acid**	135.029	HMDB00613	0,836236
**2-Methylbutyroylcarnitine**	246.17	HMDB00378	0,831544
**12-Hydroxy-12-octadecanoylcarnitine**	444.369	HMDB13154	0,830441
**Taurocholic acid**	516.296	HMDB00036	0,820824
**Isoelemicin**	207.102	HMDB29867	0,820639
**Dihydrolipoate**	207.05	HMDB12210	0,820224
**(alpha-D-mannosyl)7-beta-D-mannosyl-diacetylchitobiosyl-L-asparagine, isoform B (protein)**	234.961	HMDB62252	0,817224
**Phosphoserine**	186.016	HMDB00272	0,811223
**3-(3,4,5-Trimethoxyphenyl)propanoic acid**	239.092	HMDB30254	0,808955
**Glutamylalanine**	219.098	HMDB03764	0,80435
**N-Methylcalystegine B2**	188.092	HMDB36604	0,77938
**Histidinyl-Isoleucine**	267.144	HMDB28888	0,778403
**Pipecolic acid**	130.086	HMDB00070	0,77376
**L-Threonine**	120.066	HMDB00167	0,773686
**Decanoylcarnitine**	316.248	HMDB00651	0,770765
**Ornithine**	133.097	HMDB00214	0,758882
**Citric acid**	191.019	HMDB00094	0,75466
**6-Methylquinoline**	144.081	HMDB33115	0,752539
**Durupcoside B**	464.254	HMDB30976	0,748659
**Succinyladenosine**	384.115	HMDB00912	0,746469
**N1-Acetylspermidine**	188.176	HMDB01276	0,74626

**Cinitapride**	403.233	HMDB15698	0,745467
**3-(Acetylthio)-2-methylfuran**	79.0185	HMDB32160	0,741323
**2-Pyrrolidinone**	86.0606	HMDB02039	0,739414
**D-Glucose**	181.072	HMDB00122	0,739399
**Isobutyrylglycine**	146.081	HMDB00730	0,735231
**Glucosamine-1P**	260.053	HMDB01109	0,727695
**Gamma Glutamylglutamic acid**	277.103	HMDB11737	0,725478
**Phosphohydroxypyruvic acid**	184.986	HMDB01024	0,724684
**2-Hydroxymyristoylcarnitine**	388.306	HMDB13166	0,720521
**Pyroglutamic acid**	130.05	HMDB00267	0,71765
**Flupenthixol**	437.193	HMDB15013	0,704809
**L-Aspartic acid**	132.029	HMDB00191	0,699801
**Hexanoylglycine**	172.097	HMDB00701	0,698426
**cis-Aconitic acid**	173.008	HMDB00072	0,695489
**Homovanillic acid**	181.05	HMDB00118	0,6926
**Pentadecanoylcarnitine**	386.326	HMDB62517	0,689707
**4-Guanidinobutanoic acid**	146.093	HMDB03464	0,689601
**Benzothiazole**	136.023	HMDB32930	0,687216
**Glycine**	76.04	HMDB00123	0,681152
**3-Methylglutarylcarnitine**	290.16	HMDB00552	0,679099
**Ammonium peroxydisulfate**	226.965	HMDB37638	0,678327
**O-Demethylfonsecin**	275.054	HMDB33649	0,676863
**Deoxycytidine**	228.098	HMDB00014	0,676779
**Indolelactic acid**	204.066	HMDB00671	0,676476
**LysoPC(18:3(6Z,9Z,12Z))**	518.322	HMDB10387	0,673194
**Zapotin**	343.115	HMDB29461	0,669919
**2-Hydroxybutyric acid**	103.039	HMDB00008	0,669885
**4-Thialysine**	83.0381	HMDB29178	0,663878
**Isoacitretin**	325.184	HMDB03039	0,662896
**(S)-Isowillardiine**	200.068	HMDB30376	0,660535
**Norophthalmic acid**	276.119	HMDB05766	0,651114
**5-Acetylamino-6-formylamino-3-methyluracil**	225.061	HMDB11105	0,650626
**3-Hydroxy-cis-5-tetradecenoylcarnitine**	386.29	HMDB13330	0,650235
**Pantothenic acid**	220.118	HMDB00210	0,64752
**Meprobamate**	219.134	HMDB14515	0,64715
**Methyl (9Z)-10'-oxo-6,10'-diapo-6-carotenoate**	311.169	HMDB31976	0,644891
**3, 5-Tetradecadiencarnitine**	368.28	HMDB13331	0,641433
**Benzoic acid**	123.044	HMDB01870	0,638064
**2-Hexenoylcarnitine**	258.17	HMDB13161	0,63459
**1,3,5-Trihydroxy-10-methylacridone**	256.059	HMDB41468	0,633363
**([illegible])-Metalaxyl**	280.154	HMDB31802	0,626213
**3-(3-Hydroxyphenyl)propanoic acid**	165.055	HMDB00375	0,623728
**D-Erythrose 4-phosphate**	201.016	HMDB01321	0,622294
**L-isoleucyl-L-proline**	229.155	HMDB11174	0,621977
**1-Butylamine**	74.0971	HMDB31321	0,621876
**3-Hydroxy-6,8-dimethoxy-7(11)-eremophilen-12,8-olide**	309.174	HMDB40756	0,619669
**LysoPC(18:0)**	524.371	HMDB10384	0,618178
**L-Histidine**	154.061	HMDB00177	0,612274
**Fumaric acid**	115.002	HMDB00134	0,612105
**L-2-Amino-5-hydroxypentanoic acid**	134.081	HMDB31658	0,608407
**Spermic acid 2**	233.15	HMDB13075	0,607225
**Zymonic acid**	159.028	HMDB31210	0,601689
**Homo-L-arginine**	189.135	HMDB00670	0,601499
**3,5,6-Trihydroxy-5-(hydroxymethyl)-2-methoxy-2-cyclohexen-1-one**	205.068	HMDB41031	0,600432
**Pyridoxamine**	169.095	HMDB01431	0,599498
**2,3-Diaminosalicylic acid**	169.059	HMDB13159	0,594786
**2-Furoylglycine**	170.042	HMDB00439	0,590499
**Tetrahydropentoxyline**	367.15	HMDB29992	0,586858

**N-Acetylneuraminic acid**	308.099	HMDB00230	0,583551
**Trifluoroacetic acid**	112.984	HMDB14118	0,581749
**4-Aminobiphenyl**	170.097	HMDB13195	0,579898
**Dracunculin**	221.042	HMDB30789	0,579183
**Fexofenadine**	500.279	HMDB05030	0,576125
**4-Methoxyphenylethanol sulfate**	249.046	HMDB29229	0,574918
**Pyruvic acid**	87.0073	HMDB00243	0,573194
**3'-Hydroxyhexobarbital**	253.118	HMDB13940	0,571425
**2-Furancarboxaldehyde**	97.0289	HMDB32914	0,570307
**Erinapyrone C**	187.058	HMDB41026	0,568303
**Thiomorpholine 3-carboxylate**	148.043	HMDB59611	0,567885
**Phloretin**	273.08	HMDB03306	0,567562
**Cedryl acetate**	265.212	HMDB35910	0,567103
**Asymmetric dimethylarginine**	203.15	HMDB01539	0,564187
**Gamma-linolenyl carnitine**	422.327	HMDB06318	0,564083
**4-Hydroxy-2-butenoic acid gamma-lactone**	85.029	HMDB32330	0,552483
**Sphingosine**	300.29	HMDB00252	0,549955
**Fomepizole**	83.061	HMDB15344	0,539807
**Histamine**	112.087	HMDB00870	0,533286
**Mitotane**	316.948	HMDB14786	0,532122
**Xanthosine**	283.068	HMDB00299	0,529921
**Arginyl-Proline**	272.172	HMDB28717	0,529196
**Glyoxylic acid**	72.9916	HMDB00119	0,526732
**1-Arachidonoylglycerophosphoinositol**	619.289	HMDB61690	0,523828
**5-Hydroxymethyl-4-methyluracil**	157.061	HMDB00544	0,521406
**3-Methylene-indolenine**	130.063	HMDB11664	0,520481
**Octadecanamide**	284.295	HMDB34146	0,519262
**Oxoglutaric acid**	147.031	HMDB00208	0,510612
**Quinone**	109.029	HMDB03364	0,510298
**Arabinonic acid**	165.039	HMDB00539	0,510254
**Monoisobutyl phthalic acid**	223.097	HMDB02056	0,509122
**Sphinganine**	302.305	HMDB00269	0,504593
**L-beta-aspartyl-L-leucine**	247.129	HMDB11166	0,504166
**Acetyl-N-formyl-5-methoxykynurenamine**	263.104	HMDB04259	0,502465
**Aspartyl-Histidine**	269.088	HMDB28755	0,499879
**Erythro-5-hydroxy-L-lysinium(1+)**	164.117	HMDB62570	0,499849
**Deoxyinosine**	251.077	HMDB00071	0,498243
**trans-2-Dodecenoylcarnitine**	342.264	HMDB13326	0,496868
**(ent-2b,4S,9a)-2,4,9-Trihydroxy-10(14)-oplopen-3-one 2-(2-methylbutanoate) 9-(3-methyl-2E-pentenoate)**	449.287	HMDB40993	0,496129
**Cinnamic acid**	149.06	HMDB00567	0,495998
**(S)-3,4-Dihydroxybutyric acid**	119.034	HMDB00337	0,495077
**Thorium**	233.042	HMDB29215	0,495014
**S-Acetyldihydrolipoamide**	250.094	HMDB01526	0,494141
**Asparaginyl-Isoleucine**	246.145	HMDB28734	0,493467
**5,6,7,8-Tetrahydro-4-methylquinoline**	148.112	HMDB29709	0,491869
**4-Pyridoxic acid**	184.058	HMDB00017	0,48913
**Tiglylcarnitine**	244.154	HMDB02366	0,483509
**Formiminoglutamic acid**	173.056	HMDB00854	0,482806
**Orotidine**	287.052	HMDB00788	0,478949
**N-(3-Methylbutyl)acetamide**	116.107	HMDB31651	0,478408
**(1R,3R,4R,5S,6S,8x)-1-Acetoxy-8-angeloyloxy-3,4-epoxy-5-hydroxy-7(14),10-bisaboladien-2-one**	405.192	HMDB39971	0,475249
**N-Acetylvanilalanine**	252.088	HMDB11716	0,474226
**Methocarbamol**	242.1	HMDB14567	0,472488
**Methionyl-Threonine**	251.103	HMDB28983	0,47117
**2-Aminomuconic acid**	158.045	HMDB01241	0,469803
**Uracil**	113.035	HMDB00300	0,468658
**N'-Formylkynurenine**	237.087	HMDB01200	0,462608

**2,4,6-Octatriyn-1-ol**	119.049	HMDB30968	0,459115
**Chlorohydrin**	109.023	HMDB35190	0,458711
**2,8-Dihydroxyadenine**	166.038	HMDB00401	0,458449
**Isoprothiolane**	291.07	HMDB31779	0,457873
**Glutarylglycine**	188.056	HMDB00590	0,457184
**3-Dehydroxycarnitine**	146.118	HMDB06831	0,454728
**1-Methylhistidine**	170.093	HMDB00001	0,452375
**5-Aminopentanal**	102.092	HMDB12815	0,45122
**Dihydro-4-mercapto-3(2H)-furanone**	119.016	HMDB39786	0,45094
**N-Ornithyl-L-taurine**	240.102	HMDB33519	0,449861
**6-Feruloylglucose 2,3,4-trihydroxy-3-methylbutylglycoside**	238.091	HMDB36214	0,447969
**Aspartyl-L-proline**	231.098	HMDB02335	0,446086
**3-Sulfinoalanine**	152.001	HMDB00996	0,445549
**Glycerol 3-phosphate**	171.005	HMDB00126	0,440815
**Coumarin**	147.044	HMDB01218	0,438759
**Prostaglandin F1a**	357.262	HMDB02685	0,438351
**Glycyl-Phenylalanine**	223.108	HMDB28848	0,438265
**N-Acetylornithine**	175.108	HMDB03357	0,437715
**Succinic anhydride**	101.024	HMDB32523	0,436918
**Lofexidine**	257.028	HMDB15606	0,43447
**Trifluoromethyl-bismethyl ketone**	71.0298	HMDB61928	0,43216
**Pyridine**	80.0501	HMDB00926	0,431432
**Kamahine C**	267.124	HMDB38935	0,426584
**Vorinostat**	265.155	HMDB15568	0,42391
**(S)C(S)S-S-Methylcysteine sulfoxide**	152.036	HMDB29432	0,419726
**Trimethylamine N-oxide**	76.0764	HMDB00925	0,418566
**Glycylprolylhydroxyproline**	286.144	HMDB02171	0,418389
**2-Hydroxy-3-methylpentanoic acid**	131.07	HMDB00317	0,415475
**3-Hydroxyhexadecadienoylcarnitine**	412.306	HMDB13335	0,41402
**2,2-dichloro-1,1-ethanediol**	130.965	HMDB62193	0,411393
**13-L-Hydroperoxylinoleic acid**	313.237	HMDB03871	0,410271
**1-(2,3-Dihydro-1H-pyrrolizin-5-yl)-1,4-pentanedione**	206.117	HMDB40046	0,409167
**Glutaconic acid**	129.018	HMDB00620	0,406239
**Canrenone**	339.2	HMDB03033	0,405598
**Glycylproline**	173.092	HMDB00721	0,40499
**2-Ketobutyric acid**	101.023	HMDB00005	0,40331
**5-Hydroxylysine**	163.11	HMDB00450	0,401736
**Magnesium Sulfate**	118.929	HMDB14791	0,400846
**Toxin T2 tetrol**	299.148	HMDB36159	0,397572
**L-Agaridoxin**	255.098	HMDB29445	0,397471
**Indoxyl sulfate**	212.002	HMDB00682	0,396788
**1-Isothiocyanatobutane**	116.053	HMDB31328	0,394638
**Alanyl-Proline**	187.108	HMDB28695	0,39459
**N-Acetylhistidine**	196.074	HMDB32055	0,392507
**L-gamma-glutamyl-L-isoleucine**	261.145	HMDB11170	0,391282
**1-Methyladenosine**	282.119	HMDB03331	0,391124
**Liqcoumarin**	217.048	HMDB29518	0,389805
**(1'R)-Nepetalic acid**	185.115	HMDB36117	0,388907
**1,3,11-Tridecatriene-5,7,9-triyne**	167.084	HMDB34294	0,38854
**N-Stearoyl tyrosine**	448.342	HMDB62343	0,386684
**Hexylamine**	102.128	HMDB32323	0,38591
**Arachidonic acid**	305.247	HMDB01043	0,383897
**Benzaldehyde**	107.05	HMDB06115	0,381844
**3a,7a,12b-Trihydroxy-5b-cholanoic acid**	407.284	HMDB00312	0,379739
**2-Diethylaminoethanol**	118.123	HMDB33971	0,379062
**Castavinol**	549.168	HMDB29808	0,378224
**Allixin**	227.126	HMDB40705	0,375349
**L-Serine**	104.034	HMDB00187	0,374837
**Aldosterone**	361.201	HMDB00037	0,374241

**1-Isothiocyanato-3-phenylpropane**	176.055	HMDB38444	0,373976
**(2R,3R,4R)-2-Amino-4-hydroxy-3-methylpentanoic acid**	148.097	HMDB29449	0,37193
**Glyceric acid**	105.018	HMDB00139	0,368375
**S-(Allylthio)-L-cysteine**	194.033	HMDB38669	0,367234
**5-Hydroxyindoleacetic acid**	192.065	HMDB00763	0,365973
**3-Hydroxycapric acid**	187.133	HMDB02203	0,364507
**Putrescine**	89.1079	HMDB01414	0,362615
**4'-Hydroxyclobazam**	315.059	HMDB60771	0,362492
**Urocanic acid**	139.05	HMDB00301	0,360576
**S-Cysteinosuccinic acid**	236.023	HMDB29418	0,360406
**N-lactoyl-Tryptophan**	275.104	HMDB62178	0,36011
**Lucidenic acid M**	463.303	HMDB35973	0,359889
**Wasalexin A**	293.043	HMDB34765	0,359524
**Emtricitabine**	246.038	HMDB15017	0,359008
**Phenylalanylphenylalanine**	313.154	HMDB13302	0,358407
**8-Hydroxy-5,6-octadienoic acid**	157.084	HMDB31101	0,355539
**N-Acetylputrescine**	131.118	HMDB02064	0,352938
**Sinapyl alcohol**	211.097	HMDB13070	0,352875
**5-Hydroxy-2-furoic acid**	127.002	HMDB59784	0,35205
**Oxalosuccinic acid**	189.002	HMDB03974	0,35019
**(-)-erythro-Anethole glycol 1-glucoside**	343.14	HMDB33066	0,349764
**Lysyl-Tryptophan**	331.176	HMDB28962	0,348595
**Cytosine**	112.051	HMDB00630	0,348285
**1,3,7-Trimethyluric acid**	209.066	HMDB02123	0,347447
**Dihydrovaltrate**	425.215	HMDB34492	0,346613
**Indole**	118.065	HMDB00738	0,346375
**7-Hydroxyoctanoic acid**	159.102	HMDB00486	0,34637
**Gerberinol**	365.105	HMDB33304	0,345033
**Pirbuterol**	241.155	HMDB15407	0,34433
**Prazepam**	325.113	HMDB15527	0,342993
**4-Hydroxyproline**	132.066	HMDB00725	0,342562
**LysoPC(20:4(5Z,8Z,11Z,14Z))**	544.339	HMDB10395	0,340499
**Phthalic acid**	167.032	HMDB02107	0,338523
**2-Acetylthiazole**	126.002	HMDB32964	0,336651
**Carissanol**	377.157	HMDB30081	0,336136
**Dimethylallylpyrophosphate**	247.013	HMDB01120	0,335953
**3-Dehydroquinate**	189.04	HMDB12710	0,335825
**Uridine**	245.077	HMDB00296	0,335794
**Finasteride**	187.144	HMDB01984	0,335347
**2-phosphonato-D-glycerate(3-)**	181.965	HMDB62707	0,335038
**Deoxycholic acid glycine conjugate**	448.307	HMDB00631	0,334603
**(S)-Reticuline**	330.17	HMDB03601	0,333141
**Vanillactic acid**	213.075	HMDB00913	0,331174
**3-Hydroxy-2-methylpyridine-4,5-dicarboxylate**	196.028	HMDB06955	0,329989
**Glutaminyl-Isoleucine**	260.161	HMDB28800	0,328322
**1-Isothiocyanato-7-(methylthio)heptane**	204.087	HMDB38440	0,326609
**S-(2-carboxypropyl)-Cysteamine**	164.074	HMDB02169	0,322331
**Palmitoyl glucuronide**	419.301	HMDB10331	0,321019
**N-Acetylserotonin**	219.113	HMDB01238	0,320448
**Norfuraneol**	113.023	HMDB31859	0,320244
**Cyanidin-3-galactoside**	243.045	HMDB29195	0,319348
**Asparaginyl-Proline**	230.114	HMDB28739	0,317168
**Heptanoylcarnitine**	274.201	HMDB13238	0,317044
**(+)-2,3-Dihydro-3-methyl-1H-pyrrole**	84.0814	HMDB33529	0,316943
**Androsterone sulfate**	369.174	HMDB02759	0,316472
**Modafinil**	274.092	HMDB14883	0,314921
**Indane**	119.086	HMDB59837	0,312361
**3',4'-Dihydrodiol**	287.1	HMDB13895	0,312263
**Dezocine**	246.182	HMDB15340	0,311838

**2,6-Dimethoxy-4-methylphenol**	169.086	HMDB29680	0,311386
**Loxapine**	326.109	HMDB14552	0,311283
**N-acetyltryptophan**	245.093	HMDB13713	0,308575
**Vinylacetylglycine**	144.066	HMDB00894	0,30738
**Serinyl-Valine**	205.119	HMDB29052	0,306301
**Dicumarol**	335.061	HMDB14411	0,306187
**Epinephrine**	184.095	HMDB00068	0,306155
**Tranexamic Acid**	158.118	HMDB14447	0,305513
**L-cysteine Hydrochloride**	158.003	HMDB62799	0,305171
**Stearyl citrate**	445.314	HMDB32521	0,305126
**2-Methylbutyrylglycine**	158.081	HMDB00339	0,304747
**Phenol**	95.0497	HMDB00228	0,304618
**Amyl 2-furoate**	183.102	HMDB29456	0,303357
**2-Methylcitric acid**	207.051	HMDB00379	0,303316
**Prolyl-Threonine**	217.118	HMDB29027	0,301516
**N-Acetylgalactosamine**	220.082	HMDB00212	0,299517
**Amidosulfonic acid**	97.9918	HMDB34830	0,298202
**2-Hydroxyethanesulfonate**	124.99	HMDB03903	0,296578
**Pravastatin**	425.25	HMDB05022	0,296109
**Dehydroepiandrosterone sulfate**	367.158	HMDB01032	0,296044
**2,4-Hexadienyl acetate**	141.091	HMDB32311	0,294837
**Ascorbigen**	304.08	HMDB29839	0,294199
**Ornithinium(1+)**	134.106	HMDB62718	0,292581
**L,L-Cyclo(leucylprolyl)**	211.144	HMDB34276	0,291363
**5-hydroxy-2-oxo-4-ureido-2,5-dihydro-1H-imidazole-5-carboxylate**	203.04	HMDB59663	0,289736
**Casimiroin**	232.059	HMDB33349	0,289574
**p-Cresol glucuronide**	285.1	HMDB11686	0,289249
**LysoPE(0:0/20:1(11Z))**	508.34	HMDB11482	0,288256
**Diflubenzuron**	311.04	HMDB31778	0,286856
**Isepamicin**	570.306	HMDB41911	0,286776
**Monoethyl malonic acid**	131.034	HMDB00576	0,28644
**N,N-Dimethylaniline**	122.097	HMDB01020	0,285125
**2-Isopropyl-3,5-dimethoxy-6-methylpyrazine**	197.129	HMDB29741	0,284175
**3-Ethylpyridine**	108.081	HMDB29734	0,283244
**Acetylcarnosine**	269.126	HMDB12881	0,281354
**5,8-Dihydro-6-(4-methyl-3-pentenyl)-1,2,3,4-tetrathiocin**	265.023	HMDB38142	0,281278
**1,3-Dimethyluracil**	141.066	HMDB02144	0,280609
**3-Methoxybenzenepropanoic acid**	181.086	HMDB11751	0,280179
**Hexobarbital**	237.123	HMDB15444	0,279731
**Cadmium**	112.895	HMDB03638	0,279502
**N-Acetyl-L-phenylalanine**	206.082	HMDB00512	0,279186
**Arachidyl carnitine**	456.406	HMDB06460	0,278713
**Phenol sulphate**	172.99	HMDB60015	0,278286
**Diethanolamine**	106.087	HMDB04437	0,277395
**3-Hydroxydodecanoic acid**	215.165	HMDB00387	0,27675
**L-Aspartyl-L-phenylalanine**	281.113	HMDB00706	0,276261
**3-Methylindole**	132.081	HMDB00466	0,275242
**Diaminopimelic acid**	191.103	HMDB01370	0,274496
**Hydroxyprolyl-Isoleucine**	243.135	HMDB28866	0,274441
**p-Mentha-1,3,5,8-tetraene**	133.099	HMDB29641	0,274131
**N-Acetylgalactosamine 4-sulphate**	300.04	HMDB00781	0,273081
**Ribothymidine**	259.093	HMDB00884	0,272365
**3-Methyladipic acid**	159.065	HMDB00555	0,271209
**(9E)-10-nitrooctadecenoic Acid**	328.248	HMDB62737	0,271009
**9-Decenoylcarnitine**	314.233	HMDB13205	0,27079
**Leucyl-phenylalanine**	277.156	HMDB13243	0,270493
**Acrylamide**	72.0451	HMDB04296	0,268104
**Homocysteine**	136.043	HMDB00742	0,267061

**D-Alanyl-D-alanine**	161.092	HMDB03459	0,265271
**4-Ethylphenol**	123.081	HMDB29306	0,262775
**Nadolol**	310.201	HMDB15334	0,261697
**Isoleucyl-Tyrosine**	295.165	HMDB28919	0,260683
**Alanyl-Hydroxyproline**	203.103	HMDB28688	0,260491
**Dethiobiotin**	215.139	HMDB03581	0,259775
**Cubebinone**	429.154	HMDB33259	0,259438
**Homoarecoline**	170.118	HMDB38321	0,259189
**Propionic acid**	75.0447	HMDB00237	0,259055
**Progabide**	335.095	HMDB14975	0,258801
**2-Piperidinone**	100.076	HMDB11749	0,257056
**Carbidopa**	245.113	HMDB14336	0,256853
**N(6)-Methyllysine**	161.129	HMDB02038	0,256725
**Oleamide**	282.279	HMDB02117	0,256174
**N-Acetyl-L-tyrosine**	222.077	HMDB00866	0,255833
**4-Methyl-1-phenyl-2-pentanone**	177.128	HMDB31569	0,254456
**Glutarylcarnitine**	276.144	HMDB13130	0,25366
**Cytidine monophosphate**	322.045	HMDB00095	0,253618
**2-Octenedioic acid**	173.079	HMDB00341	0,252851
**Carbenicillin**	377.086	HMDB14717	0,251365
**5-Methylthioribose 1-phosphate**	259.001	HMDB00963	0,250569
**Garcinia acid**	207.012	HMDB31159	0,250457
**2-[(5-Methylsulfinyl)-4-penten-2-ynylidene]-1,6-dioxaspiro[4.4]non-3-ene**	249.062	HMDB32670	0,24969
**Phenylglyoxylic acid**	151.039	HMDB01587	0,249049
**Methyl methanethiosulfonate**	126.99	HMDB31832	0,248106
**Bergaptol**	203.032	HMDB13679	0,246743
**Pyrocatechol**	111.044	HMDB00957	0,246244
**2-Methylglutaric acid**	145.049	HMDB00422	0,245382
**1,2,3-Trihydroxybenzene**	125.023	HMDB13674	0,244881
**7-Hydroxy-5-(4-hydroxy-2-oxopentyl)-2-methylchromone 7-glucoside**	437.143	HMDB34698	0,244154
**2,6-Diamino-4-hydroxy-5-N-methylformamidopyrimidine**	184.085	HMDB11657	0,24338
**Phenmetrazine**	178.123	HMDB14968	0,243214
**Citrulline**	176.103	HMDB00904	0,242819
**2-Propanoylthiazole**	142.034	HMDB37168	0,242759
**Losartan**	421.156	HMDB14816	0,242277
**Allantoic acid**	177.061	HMDB01209	0,241871
**Quinic acid**	191.055	HMDB03072	0,241834
**N6,N6,N6-Trimethyl-L-lysine**	189.16	HMDB01325	0,241787
**D-Xylose**	149.044	HMDB00098	0,241732
**2-Hydroxymyristic acid**	243.196	HMDB02261	0,241378
**6-Keto-decanoylcarnitine**	330.227	HMDB13202	0,240373
**Lead**	206.972	HMDB04628	0,240216
**Cyclohexylamine**	100.112	HMDB31404	0,239036
**D-1-Piperideine-2-carboxylic acid**	128.071	HMDB01084	0,238865
**N1-Methyl-2-pyridone-5-carboxamide**	153.066	HMDB04193	0,238556
**1,2-Diacylglycerol-LD-PI-pool**	120.045	HMDB62270	0,2381
**7a,12a-Dihydroxy-3-oxo-4-cholenoic acid**	405.261	HMDB00447	0,237956
**Dexfenfluramine**	232.129	HMDB15322	0,237587
**6,7-Dimethyl-8-(1-D-ribityl)lumazine**	164.067	HMDB03826	0,236985
**Shikimic acid**	175.058	HMDB03070	0,236885
**Mukonidine**	242.079	HMDB30214	0,236556
**Isosakuranin**	447.135	HMDB29481	0,235884
**Ethyl trans-p-methoxycinnamate**	205.086	HMDB30762	0,235821
**3-Hydroxy-5, 8-tetradecadiencarnitine**	384.274	HMDB13332	0,235552
**Androsterone glucuronide**	234.132	HMDB02829	0,234673
**Cotinine methonium ion**	192.123	HMDB01365	0,234654
**2-Phosphoglyceric acid**	184.985	HMDB00362	0,234443

**Alpha-CEHC**	279.159	HMDB01518	0,2343
**2,3,5-Trimethylfuran**	111.081	HMDB29721	0,234157
**beta-D-3-[5-Deoxy-5-(dimethylarsinyl)ribofuranosyloxy]-2-hydroxy-1-propanesulfonic acid**	391.001	HMDB32686	0,233566
**Choline**	105.114	HMDB00097	0,233535
**1,7-Dimethylguanosine**	310.115	HMDB01961	0,23321
**Sulfinpyrazone sulfide**	387.115	HMDB60943	0,232496
**3-(1,1-Dimethylallyl)scopoletin 7-glucoside**	421.148	HMDB32853	0,232088
**Citicoline**	245.061	HMDB01413	0,231374
**1-Methylguanosine**	298.115	HMDB01563	0,231232
**LysoPE(0:0/22:6(4Z,7Z,10Z,13Z,16Z,19Z))**	526.293	HMDB11496	0,230299
**3-Oxooctadecanoic acid**	297.243	HMDB10736	0,229843
**3-Oxohexadecanoic acid**	269.212	HMDB10733	0,22926
**Cymorcin monoglucoside**	329.159	HMDB29777	0,228514
**Cascarillin**	407.207	HMDB36836	0,228151
**Galactitol**	181.071	HMDB00107	0,227881
**Methyl bisnorbiotinyl ketone**	215.088	HMDB04822	0,227033
**Miglitol**	206.103	HMDB14634	0,226827
**Sorbitan oleate**	429.319	HMDB29886	0,226617
**Diplosporin**	223.097	HMDB30680	0,226478
**2(N)-Methyl-norsalsolinol**	180.102	HMDB01189	0,225745
**1H-Indole-3-carboxaldehyde**	144.044	HMDB29737	0,225592
**3-Furoic acid**	111.007	HMDB00444	0,225584
**PI(16:0/22:3(10Z,13Z,16Z))**	445.288	HMDB09792	0,225522
**2,2,4,4,-Tetramethyl-6-(1-oxopropyl)-1,3,5-cyclohexanetrione**	237.116	HMDB33191	0,224723
**L-beta-aspartyl-L-alanine**	205.082	HMDB11162	0,224683
**Malonylcarnitine**	248.113	HMDB02095	0,22428
**Cadaverine**	103.123	HMDB02322	0,221904
**Methyl 2-octynoate**	155.107	HMDB31302	0,220412
**trans-3,3',4',5,5',7-Hexahydroxyflavanone**	321.06	HMDB30835	0,219917
**D-Glucuronic acid**	193.035	HMDB00127	0,219592
**9,12,13-TriHOME**	329.234	HMDB04708	0,219242
**2,3-diketogulonate**	192.024	HMDB62803	0,21648
**2-Octenoic acid**	141.091	HMDB00392	0,215812
**1-(1-Propenylthio)propyl propyl disulfide**	223.064	HMDB33041	0,215371
**cis-4-Decenedioic acid**	199.097	HMDB00603	0,215316
**3-Aminopropionaldehyde**	74.0607	HMDB01106	0,214759
**7-Methylguanine**	166.073	HMDB00897	0,214208
**Docosanamide**	340.358	HMDB00583	0,213605
**Azelaic acid**	187.097	HMDB00784	0,213455
**Homocitrulline**	190.119	HMDB00679	0,213147
**Stearidonyl carnitine**	420.31	HMDB06463	0,213042
**3-Hydroxyisoheptanoic acid**	145.086	HMDB02207	0,212542
**N-Acetyl-S-(N-methylcarbamoyl)cysteine**	219.045	HMDB41942	0,212115
**6-Dimethylaminopurine**	162.076	HMDB00473	0,211978
**5'-(3',4'-Dihydroxyphenyl)-gamma-valerolactone sulfate**	289.033	HMDB29191	0,210825
**Carboxyphosphamide**	291.008	HMDB60449	0,210804
**Glycerol tripropanoate**	261.131	HMDB32857	0,210443
**Acetylphosphate**	140.996	HMDB01494	0,210335
**Guanidinosuccinic acid**	176.066	HMDB03157	0,209922
**Ecgonine methyl ester**	200.128	HMDB06406	0,20991
**3-Bromosulfolane**	198.94	HMDB40457	0,209435
**3,4,5-Trimethoxycinnamic acid**	239.09	HMDB02511	0,20898
**Alpha-ketoisovaleric acid**	115.039	HMDB00019	0,208811
**2-Methyl-2-cyclopenten-1-one**	97.0653	HMDB31545	0,208213
**alpha-Hydroxymetoprolol**	284.186	HMDB60994	0,207985
**Suberic acid**	173.081	HMDB00893	0,206208
**Potassium 2-(1'-ethoxy) ethoxypropanoate**	199.037	HMDB37192	0,205702

**Allantoin**	157.036	HMDB00462	0,205391
**(R)-N-Methylsalsolinol**	194.118	HMDB03626	0,205235
**2-Amino-3,4-dimethylimidazo[4,5-f]quinoline**	213.112	HMDB29707	0,204682
**Cortisol**	363.217	HMDB00063	0,203878
**Metenamine**	141.114	HMDB29598	0,203834
**(S)-9-Hydroxy-10-undecenoic acid**	201.148	HMDB32662	0,203571
**1-Cyano-2-hydroxy-3-butene**	98.0606	HMDB31339	0,20316
**Sertraline**	304.071	HMDB05010	0,203085
**Osmundalactone**	129.055	HMDB31303	0,202332
**alpha-(1,2-Dihydroxyethyl)-1,2,3,4-tetrahydro-7-hydroxy-9-methoxy-3,4-dioxocyclopenta[c][1]benzopyran-6-acetaldehyde**	347.075	HMDB62442	0,201546
**Methyl (7Z,9Z,9'Z)-6'-apo-y-caroten-6'-oate**	473.345	HMDB31381	0,201432
**Pregnanetriol**	337.274	HMDB06070	0,201374
**Scopolamine**	302.135	HMDB03573	0,201111
**Phosphoglycolic acid**	156.991	HMDB00816	0,200671
**Docosa-4,7,10,13,16-pentaenoyl carnitine**	474.358	HMDB06321	0,200487
**PC(14:0/18:1(11Z))**	732.548	HMDB07872	0,199924
**Plastoquinone 3**	341.251	HMDB33786	0,199015
**Citranaxanthin**	457.35	HMDB36883	0,197356
**Methoxybrassinin**	267.059	HMDB33351	0,197003
**Ciclopirox**	208.133	HMDB15319	0,196955
**Oleic acid**	281.249	HMDB00207	0,195361
**Dihydro-2,4,6-tris(2-methylpropyl)-4h-1,3,5-dithiazine**	290.196	HMDB32221	0,194704
**Capryloylglycine**	200.129	HMDB00832	0,19306
**Zalcitabine**	212.103	HMDB15078	0,192929
**Chlorphenesin**	201.034	HMDB14994	0,19243
**Tetraphyllin B**	286.091	HMDB29914	0,189763
**3-Indolebutyric acid**	204.102	HMDB02096	0,189663
**Indoleacetic acid**	176.071	HMDB00197	0,189621
**Flumequine**	262.084	HMDB41895	0,188507
**Prolylhydroxyproline**	229.118	HMDB06695	0,188281
**Acidissiminin**	660.49	HMDB38619	0,18803
**Succinylacetone**	159.063	HMDB00635	0,187944
**2-Isopropyl-1,4-benzenediol**	153.091	HMDB32132	0,187594
**MG(0:0/16:0/0:0)**	331.284	HMDB11533	0,186804
**2,4-Dimethylthiazole**	114.038	HMDB32974	0,186354
**L-3-Aminodihydro-2(3H)-furanone**	102.055	HMDB29387	0,18623
**Vanylglycol**	185.081	HMDB01490	0,185819
**3-Oxodecanoic acid**	185.117	HMDB10724	0,185661
**Santalyl acetate**	263.197	HMDB32501	0,185545
**Toluene**	93.0704	HMDB34168	0,183753
**Daucic acid**	203.02	HMDB31665	0,183569
**3-Butyn-1-al**	69.0342	HMDB06853	0,183442
**3-Amino-2-piperidone**	115.087	HMDB00323	0,182882
**Fluvoxamine**	319.165	HMDB14322	0,182291
**L-Fucose**	165.076	HMDB00174	0,181782
**3-Oxododecanoic acid**	213.149	HMDB10727	0,179982
**(4-Methylphenyl)acetaldehyde**	135.081	HMDB29639	0,179811
**Homocysteine thiolactone**	118.032	HMDB02287	0,179723
**Simvastatin**	419.277	HMDB05007	0,179365
**Nor-psi-tropine**	128.107	HMDB31667	0,178957
**Histidinyl-Proline**	253.127	HMDB28893	0,177674
**PE(18:4(6Z,9Z,12Z,15Z)/22:6(4Z,7Z,10Z,13Z,16Z,19Z))**	392.748	HMDB09210	0,177591
**Linoleic acid**	281.248	HMDB00673	0,177341
**Genipinic acid**	243.087	HMDB38126	0,177149
**Cyclohexanone**	97.0645	HMDB03315	0,177077
**LysoSM(d18:1)**	466.352	HMDB06482	0,176675
**Isoleucyl-Tryptophan**	318.181	HMDB28918	0,17623

**Jasmonic acid**	211.133	HMDB32797	0,176058
**Cibulins**	93.0372	HMDB30299	0,175881
**Vanilpyruvic acid**	211.058	HMDB11714	0,175763
**Erythrityl Tetranitrate**	300.991	HMDB15551	0,175639
**3-carboxy-2,3-dihydroxypropanoate**	150.014	HMDB62642	0,175458
**Delta-Hexanolactone**	115.076	HMDB00453	0,17531
**Nabam**	254.917	HMDB31224	0,175069
**3b,12a-Dihydroxy-5a-cholanoic acid**	391.289	HMDB00348	0,174884
**LysoPC(20:3(5Z,8Z,11Z))**	546.355	HMDB10393	0,174594
**Marmesin rhamnoside**	393.152	HMDB39564	0,173562
**Nookatone**	219.175	HMDB13687	0,172998
**2-(2-Thienylmethylene)-1,6-dioxaspiro[4.4]non-3-ene**	219.051	HMDB34857	0,171869
**5-Dodecenoic acid**	199.169	HMDB00529	0,171459
**Propofol**	177.127	HMDB14956	0,171242
**(R)-3-Hydroxy-5-phenylpentanoic acid**	193.086	HMDB31517	0,171234
**L-Menthyl acetoacetate**	239.165	HMDB32369	0,170495
**Octadecylamine**	270.315	HMDB29586	0,169947
**Dehydrophytosphingosine**	316.285	HMDB38057	0,169417
**(3R,7R)-1,3,7-Octanetriol**	163.133	HMDB33625	0,169251
**Varenicline**	212.118	HMDB15398	0,168032
**(Z)-1,3-Octadiene**	109.102	HMDB61897	0,167738
**Mesuagin**	389.137	HMDB35876	0,167574
**Arecoline**	156.102	HMDB30353	0,167573
**Ribitol**	151.06	HMDB00508	0,167428
**lysoPC(6:0)**	356.185	HMDB29207	0,166807
**Pyruvaldehyde**	71.0124	HMDB01167	0,166434
**5-Hydroxy-L-tryptophan**	221.092	HMDB00472	0,166423
**1-Methylinosine**	283.104	HMDB02721	0,165728
**Niazirin**	280.122	HMDB32807	0,165696
**Guanosine**	284.099	HMDB00133	0,164486
**Alclometasone**	409.175	HMDB14385	0,164172
**(S,E)-Zearalenone**	319.153	HMDB31752	0,162668
**3-Methylcyclopentadecanone**	239.237	HMDB34181	0,162249
**2-Hydroxyfluorene**	92.0433	HMDB13163	0,16084
**4-Methylcatechol**	125.06	HMDB00873	0,160452
**N-Carboxyethyl-g-aminobutyric acid**	174.076	HMDB02201	0,159822
**Cortolone-3-glucuronide**	541.266	HMDB10320	0,159756
**Quinolinic acid**	166.017	HMDB00232	0,159728
**4-Ketocyclophosphamide**	275.008	HMDB60686	0,159391
**L-phenylalanyl-L-proline**	263.139	HMDB11177	0,158454
**Alosetron**	295.152	HMDB15104	0,158075
**Methylarsonate**	140.951	HMDB12258	0,157761
**Mizoribine**	258.075	HMDB41934	0,15774
**Guaifenesin**	197.081	HMDB04998	0,157291
**Tetrahydrofuran**	73.0655	HMDB00246	0,156877
**Pipercyclobutanamide B**	595.289	HMDB36361	0,156858
**LysoPE(0:0/20:5(5Z,8Z,11Z,14Z,17Z))**	498.263	HMDB11489	0,156857
**Glucosamine**	180.087	HMDB01514	0,155956
**Citronellyl anthranilate**	276.199	HMDB32208	0,155703
**D-glycero-L-galacto-Octulose**	239.077	HMDB29954	0,155432
**5-Methoxytryptophol**	192.102	HMDB01896	0,155366
**2,6 Dimethylheptanoyl carnitine**	302.233	HMDB06320	0,154887
**Glycerol**	93.0552	HMDB00131	0,154758
**Diethylthiophosphate**	171.026	HMDB01460	0,153305
**3-hydroxynonanoyl carnitine**	318.228	HMDB61635	0,152824
**4-Hydroxybenzoic acid**	137.023	HMDB00500	0,15197
**Homogentisic acid**	169.047	HMDB00130	0,151555
**Bissulfine**	179.022	HMDB30000	0,151553
**2,2,7,7-Tetramethyl-1,6-dioxaspiro[4.4]nona-3,8-diene**	181.122	HMDB30007	0,151378

**3-Methylcyclopentene**	83.0861	HMDB31544	0,151348
**3,4-Dihydroxybenzylamine**	138.055	HMDB12153	0,151127
**5-Hydroxytryptophol**	178.086	HMDB01855	0,150989
**2-Bromophenol**	170.943	HMDB32059	0,150333
**2-Furanmethanol**	99.0445	HMDB13742	0,150251
**3,4-Dihydroxyphenylglycol**	169.05	HMDB00318	0,150158
**Arbutamine**	318.166	HMDB15234	0,150005
**Meperidine**	248.168	HMDB14597	0,149162
**5-O-Feruloylnigrumin**	226.583	HMDB38735	0,148292
**Selenomethionine**	198.001	HMDB03966	0,148238
**2-trans,4-cis-Decadienoylcarnitine**	312.217	HMDB13325	0,14799
**N4-Acetylcytidine**	286.103	HMDB05923	0,14793
**N-Methylcalystegine C1**	206.102	HMDB36394	0,14686
**1,3-Diacetylpropane**	127.075	HMDB29165	0,146285
**Silicic acid**	94.9794	HMDB31436	0,146095
**4-Heptenal diethyl acetal**	201.076	HMDB32306	0,145984
**Foeniculoside VIII**	349.183	HMDB33009	0,145359
**Glycolic acid**	75.0073	HMDB00115	0,144319
**L-beta-aspartyl-L-threonine**	233.078	HMDB11169	0,143914
**Undecanedioic acid**	215.128	HMDB00888	0,143765
**Butyric acid**	87.0438	HMDB00039	0,143758
**(S)-5-Diphosphomevalonic acid**	306.999	HMDB01090	0,143197
**(9Z,11E,13E,15Z)-4-Oxo-9,11,13,15-octadecatetraenoic acid**	291.194	HMDB31098	0,143178
**Dodecanedioic acid**	229.144	HMDB00623	0,143031
**O-Phosphoethanolamine**	142.026	HMDB00224	0,142718
**Imiquimod**	241.144	HMDB14862	0,142122
**Methylmalonylcarnitine**	262.129	HMDB13133	0,142016
**Cervonyl carnitine**	472.342	HMDB06510	0,141059
**Azosemide**	368.999	HMDB41831	0,140887
**Methyl sorbate**	127.076	HMDB29582	0,140853
**Polyethylene, oxidized**	243.124	HMDB32472	0,140559
**Hydroxylated lecithin**	147.042	HMDB32332	0,140258
**5-Methyl-2-thiophenecarboxaldehyde**	125.006	HMDB32431	0,140015
**2-Oxo-3-hydroxy-4-phosphobutanoic acid**	212.979	HMDB06801	0,139443
**alpha-hydroxysalmeterol**	432.28	HMDB61045	0,138889
**Propenoylcarnitine**	216.123	HMDB13124	0,138344
**I(-)**	126.904	HMDB59634	0,138317
**Riboflavin**	377.144	HMDB00244	0,138213
**6-Dehydrotestosterone glucuronide**	463.232	HMDB10337	0,137973
**Tocainide**	193.132	HMDB15189	0,13731
**2,3,6-Trimethylphenol**	137.098	HMDB29667	0,136851
**S-2-Propenyl 1-propenesulfinothioate**	163.026	HMDB32750	0,135255
**(S)-Spirobrassinin**	251.034	HMDB35974	0,135138
**Oxprenolol**	266.173	HMDB15520	0,135128
**p-Cresol**	109.065	HMDB01858	0,135118
**Porphobilinogen**	227.103	HMDB00245	0,135038
**11-Hydroxy-12-methoxydihydrokawain**	279.12	HMDB30766	0,134992
**7-Ketodeoxycholic acid**	405.268	HMDB00391	0,134506
**([illegible])-3-Hydroxynonanoic acid**	173.117	HMDB31513	0,134425
**Cassiachromone**	233.079	HMDB30813	0,13426
**LysoPC(18:2(9Z,12Z))**	520.333	HMDB10386	0,133747
**2,4-Dihydroxy-6,7-dimethoxy-2H-1,4-benzoxazin-3(4H)-one**	240.049	HMDB37548	0,132866
**(-)-trans-Carveol glucoside**	313.164	HMDB29848	0,132694
**1,1'-(Tetrahydro-6a-hydroxy-2,3a,5-trimethylfuro[2,3-d]-1,3-dioxole-2,5-diyl)bis-ethanone**	257.103	HMDB32527	0,132654
**trans-trans-Muconic acid**	141.018	HMDB02349	0,132647
**Tetradecanedioic acid**	257.176	HMDB00872	0,132498
**8-O-Methyloblongine**	327.18	HMDB34579	0,132354
**Fenthion**	279.029	HMDB33209	0,132124

**4-Aminophenol**	110.06	HMDB01169	0,132065
**1-Methyl-1,3-cyclohexadiene**	95.0861	HMDB31532	0,131971
**3-hydroxyundecanoyl carnitine**	346.259	HMDB61637	0,131762
**Alpha-Linolenic acid**	279.232	HMDB01388	0,130933
**Vitamin K1 2,3-epoxide**	467.358	HMDB02972	0,130763
**(2xi,6xi)-7-Methyl-3-methylene-1,2,6,7-octanetetrol**	203.128	HMDB33217	0,130526
**Avenic acid A**	323.147	HMDB30416	0,130201
**Melibiose**	341.109	HMDB00048	0,130116
**2-Propene-1-thiol**	75.0267	HMDB31635	0,130014
**Dityrosine**	359.125	HMDB06045	0,129805
**Chondrillasterol 3-[glucosyl-(1->2)-glucosyl-(1->2)-glucoside]**	450.274	HMDB33887	0,129442
**Cordeauxione**	293.063	HMDB34310	0,129189
**Glutaminyl-Glutamate**	275.11	HMDB28796	0,128527
**Sertindole**	441.189	HMDB15618	0,127675
**(E)-2-octenal**	127.112	HMDB13809	0,127577
**Ethyl glucuronide**	223.085	HMDB10325	0,127427
**Menadiol dibutyrate**	315.157	HMDB32726	0,127052
**Glycyl-Tryptophan**	262.119	HMDB28852	0,126906
**Flurandrenolide**	437.236	HMDB14984	0,126137
**Desflurane**	166.992	HMDB15320	0,125997
**Propyl propane thiosulfonate**	227.091	HMDB32496	0,125682
**Hydrocortamate**	476.307	HMDB14907	0,125264
**(10S,11S)-Pterosin C**	235.132	HMDB30763	0,125155
**3-Hydroxysebacic acid**	217.108	HMDB00350	0,124702
**2,6-Dimethoxy-4-propylphenol**	195.102	HMDB36226	0,124646
**PI(16:0/20:2(11Z,14Z))**	863.557	HMDB09786	0,124614
**Benzene**	79.0547	HMDB01505	0,123817
**Pregnenolone sulfate**	397.2	HMDB00774	0,123761
**Metyrosine**	194.081	HMDB14903	0,121544
**Betamethasone**	393.21	HMDB14586	0,121014
**Coriandrone D**	353.158	HMDB29972	0,120967
**Pteroside B**	381.188	HMDB30760	0,120581
**2-(3-carboxy-3-(trimethylammonio)propyl)-L-histidine**	300.181	HMDB59617	0,120055
**Melphalan**	305.086	HMDB15176	0,119978
**2,3-Dihydroxyvaleric acid**	133.049	HMDB00421	0,119737
**Clenbuterol**	275.076	HMDB15477	0,119713
**8-Acetylegelolide**	309.131	HMDB37772	0,119021
**Indole-3-carbinol**	148.076	HMDB05785	0,118909
**Berberine**	337.126	HMDB03409	0,118683
**Cysteinyl-Cysteine**	225.035	HMDB28772	0,118673
**Citronellyl beta-sophoroside**	481.262	HMDB32839	0,118648
**Asparaginyl-Hydroxyproline**	246.108	HMDB28732	0,11857
**(R)-3-Hydroxy-Octadecanoic acid**	299.259	HMDB10737	0,118406
**Gentisic acid**	153.018	HMDB00152	0,118262
**6,11-Dihydroxy-2,2-dimethylpyrano[3,2-c]xanthen-7(2H)-one**	311.089	HMDB40357	0,118099
**2-Aminoacrylic acid**	88.0399	HMDB03609	0,116965
**Tungsten**	184.96	HMDB01989	0,116644
**Gabapentin**	172.133	HMDB05015	0,116553
**Fluorouracil**	131.024	HMDB14684	0,116546
**S-Carboxymethyl-L-cysteine**	178.017	HMDB29415	0,116308
**N-Desmethylpromazine**	269.113	HMDB13939	0,116227
**Eplerenone**	413.2	HMDB14838	0,116164
**(E)-2-Butenal**	71.0498	HMDB34233	0,116132
**3'-Hydroxy-HT2 toxin**	441.21	HMDB36162	0,115243
**Dimethyl sulfone**	95.0166	HMDB04983	0,114925
**3-Indolepropionic acid**	190.086	HMDB02302	0,114497
**Acetylglycine**	116.034	HMDB00532	0,114155

**5-Hexyltetrahydro-2-oxo-3-furancarboxylic acid**	215.126	HMDB30984	0,114121
**3-Hydroxy-4-aminopyridine sulfate**	188.997	HMDB61120	0,113939
**1-(beta-D-Ribofuranosyl)-1,4-dihydronicotinamide**	257.113	HMDB11648	0,113779
**Corchorifatty acid F**	329.231	HMDB35919	0,113331
**(R)-2-Benzylsuccinate**	209.078	HMDB12127	0,112923
**2-Hydroxypyridine**	96.0449	HMDB13751	0,112871
**Gluconolactone**	177.04	HMDB00150	0,111834
**Ethambutol**	205.193	HMDB14474	0,111246
**Lorazepam**	319.002	HMDB14332	0,11057
**N-Undecanoylglycine**	244.191	HMDB13286	0,110256
**5-(2-Methylpropyl)tetrahydro-2-oxo-3-furancarboxylic acid**	185.081	HMDB30988	0,109882
**1-(alpha-Methyl-4-(2-methylpropyl)benzeneacetate)-beta-D-Glucopyranuronic acid**	383.168	HMDB10343	0,109545
**D-Glyceraldehyde 3-phosphate**	171.006	HMDB01112	0,109378
**Hydroxyphenylacetylglycine**	208.06	HMDB00735	0,108777
**4-Hydroxypropofol**	195.138	HMDB14018	0,108555
**7(14)-Bisabolene-2,3,10,11-tetrol**	271.192	HMDB35918	0,108163
**Methionyl-Methionine**	281.1	HMDB28979	0,107804
**L-beta-aspartyl-L-glutamic acid**	263.087	HMDB11164	0,107235
**Forasartan**	209.128	HMDB15434	0,107212
**Dolichosterone**	463.342	HMDB34336	0,107122
**Hydralazine pyruvate hydrazone**	231.084	HMDB61143	0,107094
**Benzamide**	122.06	HMDB04461	0,106965
**Methylimidazole acetaldehyde**	125.071	HMDB04181	0,106745
**Santene**	123.117	HMDB38140	0,106193
**3-Chloro-1-(4-hydroxy-3-methoxyphenyl)-1,2-propanediol**	233.059	HMDB34633	0,105833
**Schidigeragenin B**	429.302	HMDB35506	0,105654
**N-(1-Deoxy-1-fructosyl)phenylalanine**	326.125	HMDB37846	0,104784
**Fructose 6-phosphate**	259.022	HMDB00124	0,104755
**1,2-Benzisothiazol-3(2H)-one**	152.017	HMDB34413	0,104086
**2,3,6,7-Tetrahydro-7-methylcyclopent[b]azepin-8(1H)-one**	164.107	HMDB39661	0,104064
**Gibberellin A55**	365.157	HMDB33420	0,103741
**2-Keto-glutaramic acid**	144.029	HMDB01552	0,103707
**Pyridoxal**	168.066	HMDB01545	0,103419
**Chlorhexidine**	253.109	HMDB15016	0,102984
**2-Phenylethyl beta-D-glucopyranoside**	283.119	HMDB29819	0,102591
**1-Stearoylglycerophosphoserine**	522.284	HMDB61698	0,10256
**Polypropylene glycol (m w 1,200-3,000)**	135.102	HMDB32478	0,102247
**Glutamyl-Lysine**	275.147	HMDB28824	0,102026
**Cycloserine**	103.051	HMDB14405	0,101399
**1-(2,4,6-Trimethoxyphenyl)-1,3-butanedione**	251.092	HMDB30668	0,101363
**Cerulenin**	224.13	HMDB15168	0,101103
**Debrisoquine**	176.117	HMDB06543	0,100561
**Sebacic acid**	201.113	HMDB00792	0,10046
**beta-D-Glucopyranosyl-11-hydroxyjasmonic acid**	387.166	HMDB39964	0,100325
**Hexadecanedioic acid**	285.207	HMDB00672	0,0993983
**1,11-Undecanedicarboxylic acid**	243.16	HMDB02327	0,099126
**Triamcinolone**	393.172	HMDB14758	0,0983637
**(3beta,22E)-26,27-Dinorergosta-5,22-dien-3-ol**	371.327	HMDB29863	0,0981042
**5-Phenylvaleric acid**	179.107	HMDB02043	0,0974069
**4,4'-Thiobis-2-butanone**	175.079	HMDB37155	0,0967678
**9-Hydroxycalabaxanthone**	409.162	HMDB31920	0,0959451
**5-Ethyl-3-hydroxy-4-methyl-2(5H)-furanone**	143.07	HMDB33551	0,0958729
**Bethanidine**	178.134	HMDB14362	0,0957617
**1-Methoxy-1-(2,4,5-trimethoxyphenyl)-2-propanol**	255.122	HMDB31772	0,0956969
**12(13)Ep-9-KODE**	309.207	HMDB13623	0,0954374
**Isovalerylglutamic acid**	232.118	HMDB00726	0,0954082
**Corchoionoside B**	399.165	HMDB30975	0,0953589
**Galactosylhydroxylysine**	325.162	HMDB00600	0,0949313

**Sunitinib**	397.205	HMDB15397	0,0949266
**epsilon-Viniferin**	228.08	HMDB30604	0,0949212
**trans-4,5-epoxy-2(E)-decenal**	169.122	HMDB13105	0,0948911
**(R)-Pelletierine**	142.123	HMDB30324	0,0948115
**Otobanone**	339.12	HMDB30618	0,0947246
**Nevirapine**	267.121	HMDB14383	0,0943626
**Porson**	387.18	HMDB30810	0,0938045
**N-Nonanoylglycine**	214.144	HMDB13279	0,0937379
**Carbendazim**	192.075	HMDB31769	0,0937373
**Parabanic Acid**	112.998	HMDB62802	0,0934994
**Decitabine**	227.077	HMDB15391	0,0932818
**Salicyluric acid**	194.045	HMDB00840	0,0928555
**Guanine**	152.057	HMDB00132	0,0922906
**Methylphosphate**	110.984	HMDB61711	0,0915161
**1-Propenyl 1-(1-propenylthio)propyl disulfide**	219.033	HMDB38967	0,0913672
**Dihydro-2-methoxy-2-methyl-3(2H)-thiophenone**	147.047	HMDB40237	0,0911947
**Polyporusterone B**	477.319	HMDB38496	0,0893446
**S-2-Propenyl 1-propanesulfinothioate**	163.024	HMDB32741	0,0888471
**Neuroprotectin D1**	361.235	HMDB03689	0,0883374
**4-Hydroxy-2-oxoglutaric acid**	161.008	HMDB02070	0,0882962
**16-Oxoestrone**	283.137	HMDB00372	0,0881417
**Norcotinine**	163.086	HMDB01297	0,0881024
**Distichonic acid A**	295.115	HMDB38752	0,0880972
**Penmacric acid**	201.052	HMDB29436	0,08809
**Hydroxytyrosol**	155.07	HMDB05784	0,088026
**Phthalide**	135.044	HMDB32469	0,0879617
**Boviquinone 4**	413.266	HMDB30057	0,0874027
**Desglucocoroloside**	505.314	HMDB33709	0,0864361
**2,3-Dehydrosalvipisone**	309.153	HMDB40742	0,0864201
**N-Succinyl-L,L-2,6-diaminopimelate**	291.12	HMDB12267	0,0861827
**1b-Hydroxycholic acid**	425.289	HMDB00307	0,0856382
**p-Hydroxyphenylacetic acid**	151.039	HMDB00020	0,0852118
**trans-Ferulic acid**	193.05	HMDB00954	0,0849677
**Chloral hydrate**	162.911	HMDB60451	0,0847882
**2,3-Dinor-6-keto-prostaglandin F1 a**	341.196	HMDB02277	0,0845584
**5-[(4-Hydroxypheny)ethenyl]-2-(3-methyl-1-butenyl)-1,3-benzenediol**	295.137	HMDB30599	0,0844419
**3-Methyluric acid**	181.038	HMDB01970	0,0838787
**Chloroxine**	213.983	HMDB15373	0,0835698
**2-Chloro-5-methylmaleylacetate**	207.008	HMDB60346	0,0834839
**Lipoyllysine**	335.147	HMDB12996	0,0832959
**N-Desalkyl flurazepam**	289.05	HMDB61161	0,0831391
**p-Anisidine**	124.076	HMDB29300	0,0829851
**Hydrocinnamic acid**	151.075	HMDB00764	0,0829497
**Gentian Violet**	373.256	HMDB14550	0,0826514
**1-Pyrroline-5-carboxylic acid**	114.055	HMDB01301	0,0825673
**3-Hydroxydodecanedioic acid**	245.139	HMDB00413	0,0824737
**Leukotriene A4**	319.225	HMDB01337	0,0813787
**Arbutin**	273.097	HMDB29943	0,081259
**Methyl 2-(10-heptadecenyl)-6-hydroxybenzoate**	387.286	HMDB38523	0,081212
**MG(0:0/16:1(9Z)/0:0)**	327.254	HMDB11534	0,0811735
**Gemfibrozil**	251.161	HMDB15371	0,0808314
**13'-Carboxy-gamma-tocopherol**	447.346	HMDB12557	0,0807315
**13-Hydroxy-9-methoxy-10-oxo-11-octadecenoic acid**	343.248	HMDB40901	0,0804823
**4-Nitrophenol**	138.018	HMDB01232	0,0804275
**2,2,4,4-Tetramethyl-6-(1-oxobutyl)-1,3,5-cyclohexanetrione**	251.132	HMDB33197	0,07979
**(-)-trans-Carveol**	153.128	HMDB03450	0,0796106
**Dodecamethylpentasiloxane**	383.135	HMDB62731	0,0795961

**4-Hydroxy-3-prenylbenzoic acid glucoside**	367.139	HMDB39891	0,0795733
**Isopentenyladenine-9-N-glucoside**	364.197	HMDB12240	0,0791677
**O-methoxycatechol-O-sulphate**	205.017	HMDB60013	0,079141
**Phaseolic acid**	261.134	HMDB31897	0,0785469
**(R)-Dihydromaleimide**	98.0233	HMDB30276	0,0778666
**3-Methylcrotonylglycine**	158.081	HMDB00459	0,0776371
**alpha-Fluoro-beta-alanine**	108.045	HMDB60434	0,0776099
**Traumatic acid**	229.141	HMDB00933	0,0758664
**6-Gingesulfonic acid**	359.156	HMDB38999	0,0758278
**Allolithocholic acid**	375.294	HMDB00381	0,0758195
**Indoleacetaldehyde**	160.076	HMDB01190	0,0751403
**Neryl glucoside**	315.181	HMDB29346	0,0743013
**Aspartyl-Glutamine**	262.103	HMDB28751	0,0738087
**Sumatriptan**	296.146	HMDB05037	0,073654
**Oxalic acid**	88.9866	HMDB02329	0,0731684
**Glaucarubin**	497.236	HMDB35626	0,0730867
**threo-Syringoylglycerol**	243.086	HMDB31237	0,0730468
**Chloropentafluoroethane**	154.967	HMDB31333	0,0721315
**Oxymorphone**	302.135	HMDB15323	0,0721031
**Methyl 3-(2,3-dihydroxy-3-methylbutyl)-4-hydroxybenzoate**	253.108	HMDB32796	0,0717016
**3,4,5-Trimethoxyphenyl acetate**	225.075	HMDB31722	0,0715307
**Nicotinic acid**	124.04	HMDB01488	0,0712056
**Atenolol**	267.172	HMDB01924	0,0711442
**N-Methylphenylethanolamine**	152.107	HMDB01387	0,0708852
**4-Hydroxyphenylpyruvic acid**	179.034	HMDB00707	0,0704858
**Fucose 1-phosphate**	243.027	HMDB01265	0,0704778
**Lomustine**	234.097	HMDB15337	0,0704106
**Valproic acid glucuronide**	319.14	HMDB00901	0,0703511
**7-Acetoxy-2-methylisoflavone**	295.094	HMDB29364	0,0703281
**Phenyl vinyl sulfide**	137.041	HMDB31825	0,070179
**Tetrahydrofurfuryl butyrate**	171.102	HMDB36188	0,0693923
**Cuminaldehyde**	149.096	HMDB02214	0,0687087
**5-Nonyltetrahydro-2-oxo-3-furancarboxylic acid**	255.16	HMDB30993	0,0686841
**11-nitro-1-undecene**	200.165	HMDB62669	0,0682814
**2-Dodecylbenzenesulfonic acid**	327.201	HMDB31031	0,0681098
**15-Deacetylneosolaniol**	341.157	HMDB36157	0,0676934
**p-Cymene**	135.117	HMDB05805	0,0675947
**Anileridine**	353.222	HMDB15049	0,0673208
**Caproic acid**	117.091	HMDB00535	0,0665534
**2-(4-Methyl-5-thiazolyl)ethyl octanoate**	270.155	HMDB32423	0,0659651
**Cucurbic acid**	211.133	HMDB29388	0,0659273
**Levocabastine**	421.234	HMDB15238	0,0654754
**Prostaglandin E2**	353.231	HMDB01220	0,0652796
**Dimethylethanolamine**	90.0919	HMDB32231	0,065246
**3-Methyl-4-phenyl-3-buten-2-one**	161.096	HMDB31567	0,0650726
**Trifluridine**	297.074	HMDB14576	0,0643098
**Norepinephrine**	170.081	HMDB00216	0,0637832
**2-Phenylbutyric acid**	165.091	HMDB00329	0,0637657
**Hexaethylene glycol**	283.175	HMDB61822	0,0635475
**N-Decanoylglycine**	228.16	HMDB13267	0,0633085
**D-Limonene**	137.133	HMDB03375	0,0620937
**N-Deschlorobenzoyl indomethacin**	220.097	HMDB13988	0,06194
**3-Propylidene-1(3H)-isobenzofuranone**	175.075	HMDB31845	0,0616228
**Malonic acid**	103.002	HMDB00691	0,0606631
**3,6-Dihydro-4-(4-methyl-3-pentenyl)-1,2-dithiin**	199.061	HMDB30009	0,0606573
**Corchoionol C 9-glucoside**	385.186	HMDB29772	0,0602567
**Clozapine**	327.142	HMDB14507	0,0597853
**Dimethicone**	161.081	HMDB33532	0,0597408

**Jasmolone glucoside**	343.174	HMDB32870	0,0593304
**cis-Methylbixin**	409.236	HMDB32021	0,058687
**PE(P-16:0e/0:0)**	436.283	HMDB11152	0,0576932
**L-Cysteine**	122.027	HMDB00574	0,0568922
**Spironolactone**	415.197	HMDB14565	0,0563162
**4-Hydroxycyclohexylcarboxylic acid**	143.07	HMDB01988	0,0555263
**Armillane**	421.22	HMDB35779	0,0549709
**Octanal**	129.128	HMDB01140	0,0549198
**Senecioic acid**	101.06	HMDB00509	0,0548396
**Morellin**	273.134	HMDB30794	0,054526
**Oxoadipic acid**	159.029	HMDB00225	0,0543651
**Paxilline**	436.254	HMDB30323	0,0538385
**trans-3-Hydroxycotinine glucuronide**	369.131	HMDB01204	0,0526173
**beta-Damascenone**	191.143	HMDB13804	0,0525836
**2-Isopropylmalic acid**	175.06	HMDB00402	0,0517888
**Caprylic acid**	143.107	HMDB00482	0,0515803
**Dicyclohexyl disulfide**	231.121	HMDB41448	0,0515567
**3-Hydroxy-9-(4-hydroxyphenyl)-1H,3H-naphtho[1,8-cd]pyran-1-one**	293.078	HMDB39404	0,0514536
**3-Methyl-1-butylamine**	88.1126	HMDB31659	0,0514471
**4-Fumarylacetoacetic acid**	199.022	HMDB01268	0,051298
**Mevalonic acid**	147.065	HMDB00227	0,0499493
**Latanoprost**	433.293	HMDB14792	0,0498631
**1,4-Ipomeadiol**	169.086	HMDB30471	0,0490004
**Rishitin**	223.169	HMDB35593	0,0479272
**Histidylproline diketopiperazine**	247.118	HMDB02053	0,0478659
**Octadecanedioic acid**	313.239	HMDB00782	0,0470027
**3,4-DHPEA-EA**	379.137	HMDB29304	0,0468817
**Crotamiton**	204.138	HMDB14410	0,0467148
**p-Toluenesulfonic acid**	171.01	HMDB59933	0,0452802
**2,3-Dimethyl-2-cyclohexen-1-one**	125.096	HMDB31414	0,0452144
**Methylgingerol**	309.206	HMDB29852	0,0441857
**Tridecanoylglycine**	272.222	HMDB13317	0,0435835
**6-Acetyl-1,2,3,4-tetrahydropyridine**	126.092	HMDB30345	0,0431619
**N-Acetyl-2,3-dihydro-1H-pyrrole**	112.076	HMDB31163	0,0425068
**Lucidenic acid A**	459.28	HMDB37611	0,0424766
**12-Oxo-20-carboxy-leukotriene B4**	365.193	HMDB12550	0,0419289
**3-Hydroxyanthranilic acid**	152.034	HMDB01476	0,0417897
**(Z)-5-[(5-Methyl-2-thienyl)methylene]-2(5H)-furanone**	193.029	HMDB33845	0,0410083
**13-HDoHE**	315.264	HMDB60043	0,0407911
**Trigoforin**	189.091	HMDB29495	0,0407383
**Verimol B**	317.139	HMDB38323	0,0406362
**Glycerol 1-propanoate diacetate**	231.087	HMDB31640	0,0389345
**(Z)-[3-(Methylsulfinyl)-1-propenyl] 2-propenyl disulfide**	209.013	HMDB32653	0,0382957
**(2R*,3R*)-1,2,3-Butanetriol**	107.071	HMDB34778	0,0378536
**Metaxalone**	222.113	HMDB14798	0,0368016
**Cinncassiol D2 glucoside**	531.275	HMDB34679	0,0365703
**1-(4-Methoxyphenyl)-1-penten-3-one**	191.107	HMDB30905	0,0346418
**Dimethylbenzyl carbinyl hexanoate**	249.185	HMDB32228	0,03309
**Vanilloloside**	315.109	HMDB32013	0,0327461
**Elenolide**	223.061	HMDB30018	0,0327129
**Barbituric acid**	127.014	HMDB41833	0,0318121
**Galactosylglycerol**	253.093	HMDB06790	0,0313474
**(1R,3S,4S,6R)-6,9-Dihydroxyfenchone 6-O-b-D-glucoside**	345.156	HMDB33222	0,0304542
**Pyruvatoxime**	102.018	HMDB02455	0,0271757
**12S-HHT**	281.209	HMDB12535	0,0269099
**Phenylacetic acid**	137.06	HMDB00209	0,021672
**(Z)-1-(Methylthio)-5-phenyl-1-penten-3-yne**	189.073	HMDB32688	0,0215873
**Linocinnamarin**	341.119	HMDB30678	0,0207849

**3-[[5-Methyl-2-(1-methylethyl)cyclohexyl]oxy]-1,2-propanediol**	229.18	HMDB36133	0,0198932
**5-Aminopentanamide**	117.103	HMDB12176	0,0175487
**Methyl 7-epi-12-hydroxyjasmonate glucoside**	401.182	HMDB31763	0,0130457
**Homoveratric acid**	195.066	HMDB00434	0,00734863
